# Ecological aspects and relationships of the emblematic *Vachellia* spp. exposed to anthropic pressures and parasitism in natural hyper-arid ecosystems: ethnobotanical elements, morphology, and biological nitrogen fixation

**DOI:** 10.1007/s00425-024-04407-0

**Published:** 2024-04-25

**Authors:** Bryan Vincent, Julie Bourillon, Karine Gotty, Hassan Boukcim, Marc-André Selosse, Aurélie Cambou, Coraline Damasio, Mathis Voisin, Stéphane Boivin, Tomas Figura, Jérôme Nespoulous, Antoine Galiana, Kenji Maurice, Marc Ducousso

**Affiliations:** 1 CIRAD, UMR113 LSTM, TA A-82⁄J, Campus International de Baillarguet, 34398 Montpellier Cedex 5, France; 2 Department of Research and Development, VALORHIZ, 1900, Boulevard de la Lironde, PSIII, Parc Scientifique Agropolis, 34980 Montferrier sur Lez, France; 3 Institut Systématique Evolution Biodiversité (ISYEB), Muséum National d’Histoire Naturelle (MNHN), CNRS, Sorbonne Université, EPHE, 57 rue Cuvier, CP39, 75005 Paris, France; 4 Department of Plant Taxonomy and Nature Conservation, University of Gdańsk, Wita Stwosza 59, 80-308 Gdańsk, Poland; 5 Institut Universitaire de France, Paris, France; 6 Eco&Sols, IRD, Université de Montpellier, CIRAD, INRAE, Institut Agro, Montpellier, France; 7 Department of Mycorrhizal Symbioses, Institute of Botany, Czech Academy of Sciences, Lesní 322, Průhonice, Czech Republic; 8 Understanding Evolution Group, Naturalis Biodiversity Center, Darwinweg 2, 2333 CR, Leiden, The Netherlands

**Keywords:** Hyper-arid desert, Mistletoe, Mutualism, ^15^N natural abundance, *Retama raetam*, *Vachellia gerrardii*, *Vachellia tortilis* subsp. *raddiana*, Witch broom

## Abstract

**Main conclusion:**

Emblematic *Vachellia* spp. naturally exposed to hyper-arid conditions, intensive grazing, and parasitism maintain a high nitrogen content and functional mutualistic nitrogen-fixing symbioses.

**Abstract:**

AlUla region in Saudi Arabia has a rich history regarding mankind, local wildlife, and fertility islands suitable for leguminous species, such as the emblematic *Vachellia* spp. desert trees. In this region, we investigated the characteristics of desert legumes in two nature reserves (Sharaan and Madakhil), at one archaeological site (Hegra), and in open public domains at Al. Ward and Jabal Abu Oud. Biological nitrogen fixation (BNF), isotopes, and N and C contents were investigated through multiple lenses, including parasitism, plant tissues, species identification, plant maturity, health status, and plant growth. The average BNF rates of 19 *Vachellia gerrardii* and 21 *Vachellia tortilis* trees were respectively 39 and 67%, with low signs of inner N content fluctuations (2.10–2.63% N) compared to other co-occurring plants. The BNF of 23 *R. raetam* was just as high, with an average of 65% and steady inner N contents of 2.25 ± 0.30%. Regarding parasitism, infected *Vachellia* trees were unfazed compared to uninfected trees, thereby challenging the commonly accepted detrimental role of parasites. Overall, these results suggest that *Vachellia* trees and *R. raetam* shrubs exploit BNF in hyper-arid environments to maintain a high N content when exposed to parasitism and grazing. These findings underline the pivotal role of plant-bacteria mutualistic symbioses in desert environments. All ecological traits and relationships mentioned are further arguments in favor of these legumes serving as keystone species for ecological restoration and agro-silvo-pastoralism in the AlUla region.

**Graphical abstract:**

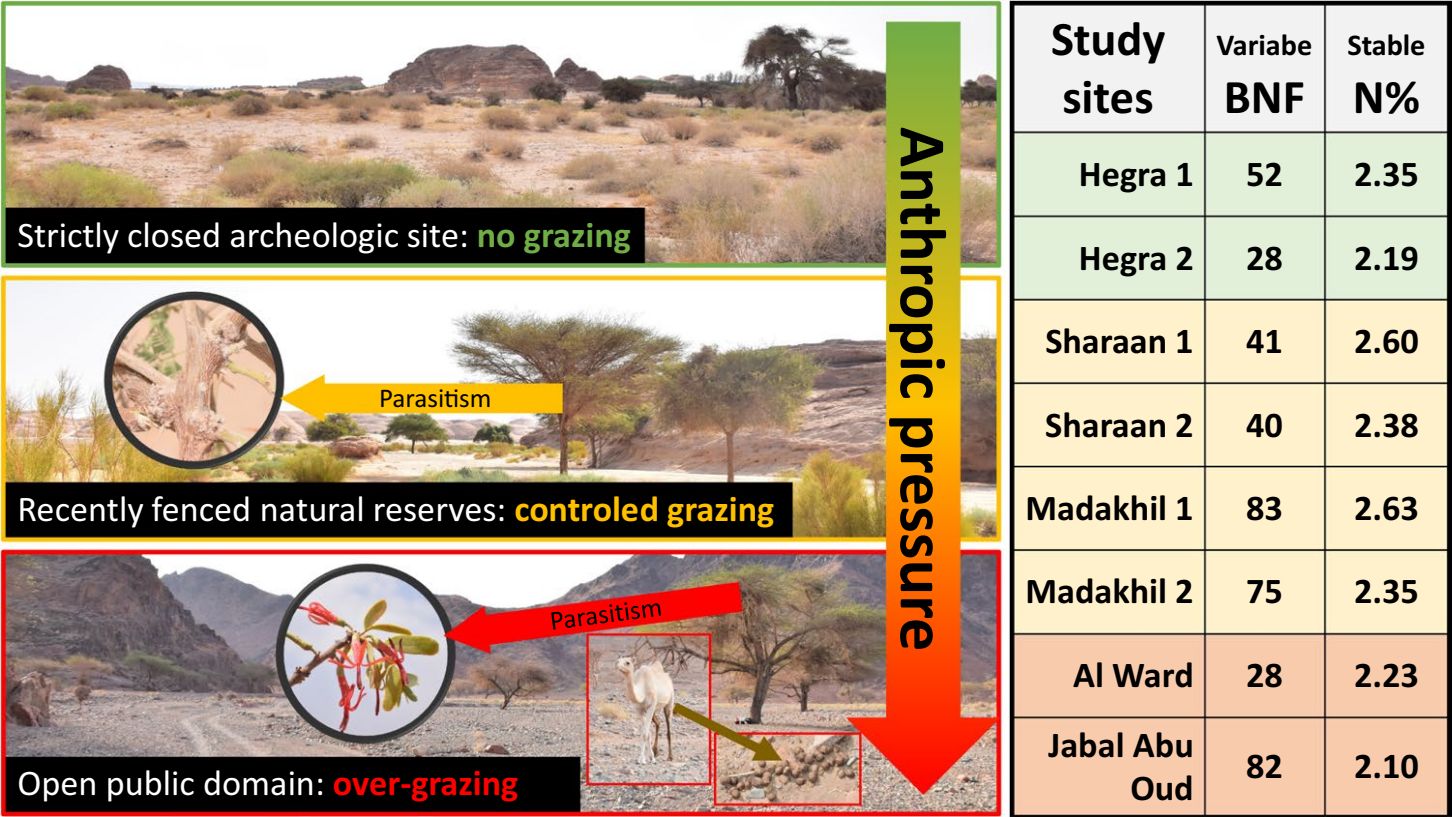

**Supplementary Information:**

The online version contains supplementary material available at 10.1007/s00425-024-04407-0.

## Introduction

Situated in the northwestern region of Saudi Arabia, AlUla is a stunning desert oasis with a rich history and natural beauty. The region is home to several archaeological sites, including the ancient city of Hegra, which was an important trade hub for the Nabataean civilization 2000 years ago and is now classified as a UNESCO World Heritage Site. The landscape of the AlUla region is characterized by sandstone formations, rocky outcrops, and mountain ranges. These ancient formations include canyons, wadis, and arches. The region has a desert climate, with hot dry conditions prevailing throughout the year. Summers are particularly harsh, with maximum day temperatures reaching 50 °C during the summer months. Winters are mild, with temperatures averaging around 20 °C in the day and dropping to around 5 °C at night. Saudi Arabia also experiences sporadic rainfall, with an average of 40 mm/year, but the pattern differs greatly between provinces and according to the time of year (Almazroui 2013; Hasanean and Almazroui 2015). The rugged terrain and the rocky landscape are also responsible for the different microclimatic conditions that prevail within the region, with some areas experiencing slightly milder temperatures than others.

The area is especially known for its date palm groves, which have been cultivated in the region for thousands of years while being a key component of the local economy (Aleid et al. 2015). In addition to date palms, AlUla region hosts rich plant species diversity, including acacia woodlands and native desert plants. The botanical review of Ansari et al. (2022) reported 45 families, 157 genera, and 227 species of angiosperms in the Tabuk region, not far from AlUla. Moreover, the vascular plant diversity in Wadi Arar in the northern part of the country was estimated to include 31 families and 196 plant species (Osman et al. 2014). Preservation of the natural beauty and ecological balance of the AlUla region is a top priority. A range of initiatives to promote sustainable tourism and protect the region’s unique flora and fauna have developed (i.e., establishment of the Sharaan Nature Reserve). This protected area is home to a wide range of plants and animals (Fig. S1) and is managed using eco-friendly practices to minimize its environmental impact. Another initiative is the 5-year SoFunLand research project that began in 2020 and is set to end in 2024. This project aims to analyze soil microbes and their functioning in arid soil to improve land use and preservation in the AlUla region.

Saudi Arabia hosts several species of *Vachellia* Wight & Arn. (ex. *Acacia*), listed in the taxonomical studies of Waly and Emad (2012). It is essential to conduct surveys of these leguminous species to support the sustainable development of agropastoral crops in desert environments. These plants—considered as being keystone species in desert ecosystems (Munzbergova and Ward 2002)—are able to fix atmospheric nitrogen (N_2_), an essential nutrient for plant growth, while improving soil fertility, offering shading to local fauna (Fig. S1i), and enhancing overall ecosystem productivity. However, AlUla region is coping with increasing anthropic pressure related to demographic expansion and the sedentarization of nomadic camel herders, thereby substantially increasing the grazing intensity in the area (Fig. S1g). The expansion of local fauna populations has also further increased the grazing pressure (Fig. S1h), a phenomenon which plays a complex and dynamic role in desert environments and is reported as having a detrimental impact on local flora in natural ecosystems of western Saudi Arabia (Al-Rowaily et al. 2015). Therefore, it is important to understand and identify a suitable balance between grazing and natural ecosystem processes to ensure sustainable land management practices. Moreover, leguminous trees in desert environments are crucial for nomads as they provide multiple ecosystem services, such as providing fuel, medicines, and fodder for cattle (Rahman et al. 2004; Hobbs et al. 2014).

In addition to grazing, leguminous trees are also exposed to infestation by mistletoe species such as *Plicosepalus acaciae* (Zucc.) Wiens & Polhill (Fig. S1d). These epiphytes survive by extracting nutrients and water from their host, hence potentially exacerbating the already existing drought stress and lack of essential nutrients in harsh environments (Schulze et al. 1984; Watling and Press 2001; Bowie and Ward 2004). Mistletoes are able to accumulate N and act as a sink, with their N contents sometimes three to four times higher than those of their host (Küppers et al. 1992; Panvini and Eickmeier 1993). The growth of this parasite is mostly dependent on the extent of N availability (Ehleringer et al. 1986), and its dispersion may be hard to control mainly because its seeds are spread through avian vectors such as yellow-vented bulbuls (*Pycnonotus xanthopygos*) (Green et al. 2009). In addition to epiphytic parasitism, *Vachellia* trees are also subjected to the Witch Broom Disease (WBD). Causal agents and factors triggering the WBD are multiple: phytoplasmas, fungi, insects, genetic mutations, viruses, etc. (Meinhardt et al. 2008; Vasilyeva et al. 2020; Al-Subhi et al. 2021; Rao et al. 2021). The symptoms manifest as an uncontrolled and local proliferation of plant tissues. The WBD consequences may be dramatic for agriculture, e.g.: loss of 50–90% of cocoa production (Meinhardt et al. 2008), and affect other tree crops, such as lime trees (Al-Subhi et al. 2021). Investigation of the in situ relationship between *Vachellia* and its parasites may provide useful information for the development and management of future ecological and restoration plans involving *Vachellia* trees.

Our study was designed to prospect multiple ecological aspects and relationships of the emblematic *Vachellia* spp. trees in the AlUla desert and to highlight the importance of mutualistic symbioses for the restoration and expansion of local ecosystems and for land use improvement. These ecological aspects include (1) the role of parasitism and anthropic pressures on the plant growth (height and width) and physiology (C and N contents), (2) ethnobotanical aspects regarding species identification, and (3) the plant ability to use nitrogen-fixing symbiose to strive in such environments. Naturally occurring populations of *Vachellia* spp. trees and their associated plant corteges (reference species) were harvested in eight regions of interest (ROI) along an anthropic/grazing gradient: (1) the strictly closed archaeological site of Hegra (fenced for over 60 years), (2) recently fenced (in 2021) nature reserves (Sharaan and Madakhil), and (3) two open areas subject to moderate and intense grazing (Jabal Abu Oud and Wady Al Ward, respectively). The parameters measured included: tree growth, and N contents, δ^13^C and δ^15^N isotopic signatures, C/N ratio, and biological nitrogen fixation (BNF). The method used to estimate the in situ BNF of *Vachellia* trees was based on the ^15^N natural abundance. *Vachellia* tree species were identified on the basis of local names, botanical descriptions, and molecular tools. The parasite:host relationship was also assessed to detect potential interference with the tree characteristics, especially with N or C contents, and BNF. Although our study is focused on *Vachellia* spp. trees, another leguminous species, *Retama raetam* (Forssk.) Webb & Berthel., was commonly found close to *Vachellia* spp. trees. Therefore, our sampling design was extended to include this second leguminous species when present and serve as a second marker/control of BNF.

## Materials and methods

### Study sites and local names of *Vachellia* trees

**Fig. 1:**
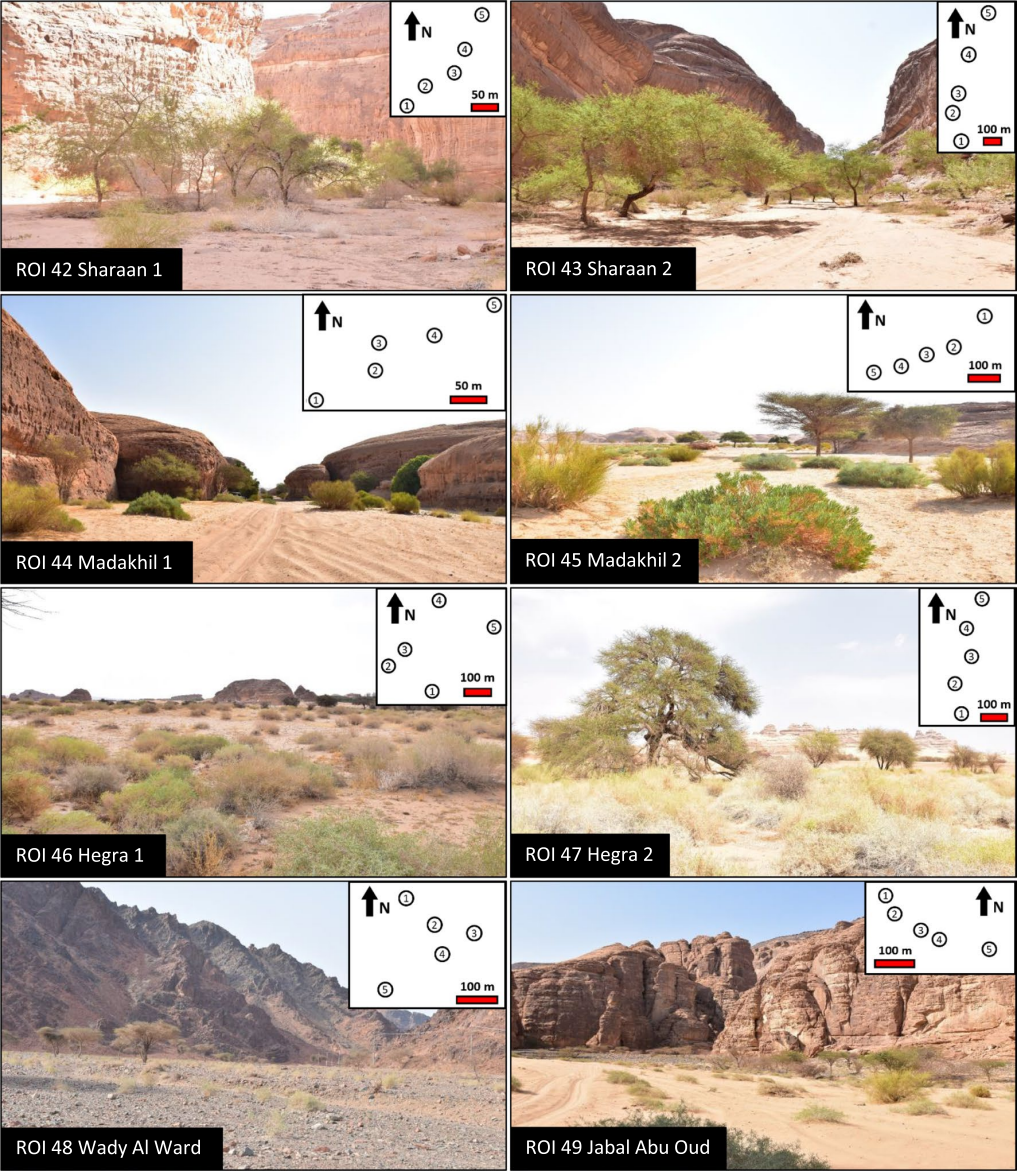
The eight regions of interest (ROI) of the study. The minimap on the top right represents the spacing of *Vachellia* spp. trees and the sampling protocol

The ancient Arabic oasis of AlUla is located in Medina Province, northwestern Saudi Arabia. A total of eight ROIs hosting *Vachellia* spp. populations was defined (Fig. 1). Study sites, tree legumes, and associated reference species are given in Table 1. Orthophotographic views of the ROIs are shown in Fig. S2. The details of the *Vachellia* trees sampled are available in Table S1.

**Table 1:** List and number of plants collected in each location and region of interest (ROI)

Location name	Sharaan		Madakhil		Hegra		Wady Al Ward	Jabal Abu Oud
Region of interest number	ROI 42	ROI 43	ROI 44	ROI 45	ROI 46	ROI 47	ROI 48	ROI 49
Reference species								
*Atriplex coriacea*	–	–	–	–	5	5	–	–
*Brachypodium retusum*	-	3	–	1	–	–	–	–
*Citrullus colocynthis*	3	1	5	5	–	–	–	1
*Ephedra aphylla*	4	1	–	1	–	–	–	2
*Fagonia bruguieri*	2	1	–	–	–	–	–	–
*Haloxylon salicornicum*	–	–	4	5	5	5	5	5
*Lycium shawii*	5	5	–	–	4	4	–	4
*Ochradenus baccatus*	5	5	2	–	3	3	–	2
*Panicum turgidum*	2	3	5	5	–	–	–	4
*Salsola baryosma*	–	–	–	–	5	5	–	–
*Suaeda vermiculata*	–	–	–	–	1	2	–	–
*Senna italica*	1	3	–	–	–	–	–	3
Parasite								
*Plicosepalus acaciae*	–	–	–	–	4	1	2	4
Witch broom disease parasite	–	–	1	3	–	–	–	–
N_2_-fixing species								
*Retama raetam*	4	5	5	5	–	–	–	4
*Vachellia* saplings	5	5	2	4	2	1	2	3
*Vachellia* trees	5	5	5	5	5	5	5	5
Anthropic pressure								
Land use	Natural reserve		Natural reserve		Archeologic site			Public domain
Grazing intensity	Low	Low	Low	Low	None	None	Very high	Moderate
Fencing (years)	2	2	2	2	70	70	None	None
Location	26°52′52.08″ N	26°53′54.89″ N	27°00′15.59″ N	27°00′20.43″ N	26°46′16.17″ N	26°47′18.29″ N	26°30′32.70″ N	26°41′15.16″ N
	38°14′18.80″ E	38°13′33.84″ E	37°47′17.12″ E	37°47′33.96″ E	37°56′24.44″ E	37°56′56.81″ E	37°37′05.12″ E	37°52′37.87″ E

Anthropic pressure includes land use, the number of years since fencing, and the grazing intensity observed in situ. Coordinates are also indicated

The first two ROIs (42 and 43) were located in the Sharaan Nature Reserve (Fig. 1). This 1500 km^2^ reserve was entirely fenced in 2021 and at the time of the study local wildlife had recently been reintroduced, thus generating low grazing pressure. The soil is mostly sandy, and *Vachellia* trees on the site are called *taleh* by local people.

The ROIs 44 and 45 were located in the natural reserve of Madakhil. Their characteristics were similar to those of ROIs 42 and 43 although they differed in the absence of wildlife reintroduction at the time of the study. The *Vachellia* trees are also called *taleh*, except for one individual (VAC 11), named *seyal*.

ROIs 46 and 47 were located inside the Hegra archeological site. Owing to its importance in human history, this area has been registered on the UNESCO World Heritage List and completely fenced off since the 1950s. Large herbivorous animals such as camels did not have any access to Hegra, so mammal grazing in the area was considered non-existent at the time of the study. *Vachellia* spp. trees sampled at these sites are called *samor* by local people, with one *seyal* individual (VAC 26).

The last two ROIs (48 and 49) were in natural ecosystems on public domain and open sites subject to moderate and intense grazing. ROI 48 was located next to Al Ward village, southwest of AlUla. The soil was highly rocky and clayey. Grazing in this area was very high, with mostly two plant species present, i.e., *Vachellia* spp. trees (locally called *seyal*) and the remains of heavily grazed *Haloxylon salicornicum* (Moq.) Bunge ex Boiss. The last ROI (49) was located in a wadi next to Jabal Abu Oud with sandy–clayey soils. The *Vachellia* tree populations were a mix of *samor* and *seyal* individuals. Despite the high grazing rate in the area, local flora was much more diverse than in ROI 48, with a total of seven non-N_2_-fixing perennial species co-occurring with *Vachellia* trees (Table 1).

### Plant sampling and preparation

Five *Vachellia* spp. were sampled in each of the eight ROI, representing a total of 40 trees (Table S1). Vegetation is scarce in this hyper-arid desert environment, so the *Vachellia* trees sampled were spaced 50–200 m from each other in each site (Fig. 1). The material collected included leaves, branches, bark, and wood of mature trees, along with saplings (less than 1 m high) when possible. Leaves and/or chlorophyllous stems of another co-occurring legume species, i.e., *Retama raetam*, were sampled when present.

Leaves of non-N_2_-fixing plant species (called reference species in this study) were also sampled and identified outside the leguminous trees, at a minimum distance of 5 m from the trunk (Table 1). Each sampled reference species was present in at least two different ROIs. The sampled reference species were *Atriplex coriacea* Forssk. (Chenopodiaceae), *Citrullus colocynthis* (L.) Schrader (Cucurbitaceae), *Ephedra aphylla* Forssk. (Ephedraceae), *Haloxylon salicornicum* (Chenopodiaceae), *Lycium shawii* Roem. & Schult. (Solanaceae), *Ochradenus baccatus* Delile (Resedaceae), *Panicum turgidum* Forssk. (Poaceae), *Salsola baryosma* (Roem. & Schult.) Dandy (Chenopodiaceae), and *Senna italica* Mill. (Fabaceae).

Parasite sampling included: (1) leaves growing on WBD infected tissues of *Vachellia*, and (2) leaves of *Plicosepalus acaciae* (Zucc.) Wiens & Polhill, a known epiphytic parasite of *Acacia *sensu lato (Fig. S1d).

All samples were placed inside 50 mL Falcon tubes with silicate beads, and finely ground (<100 µm) in a mill (Geno Grinder, SPEX SamplePrep 2010), with 1300 vertical shakes during 1 min, at the Agropolis Resource Centre for Crop Conservation, Adaptation and Diversity (ARCAD) based in Montpellier, France. Recalcitrant samples such as wood were first frozen with liquid nitrogen. Between 5 and 10 mg (dry weight) of biomass of each sample was transferred into tin capsules. Total N and C contents, ^15^N/^14^N and ^13^C/^12^C, and the C/N ratio were determined using a Thermo Flash 2000 elemental analyzer in tandem with a ThermoFinnigan DeltaV Advantage Continuous-Flow Isotope-ratio mass spectrometer.

Relative abundances of the stable isotopes (*δ* values) were calculated as follows: δ^13^C or δ^15^N = (*R*_sample_/*R*_standard_ − 1) × 1000 (‰), where *R*_sample_ is the ^13^C/^12^C or ^15^N/^14^N ratio of the sample, and *R*_standard_ is the ^13^C/^12^C ratio of the Vienna Pee Dee Belemnite standard or the ^15^N/^14^N ratio of atmospheric N_2_, respectively. Alanine served as internal standard. Primary standards were caffeine IAEA-600 (δ^13^C = −27.77 ± 0.04‰) and ammonium sulfate IAEA-N-1 (δ^15^N = 0.40 ± 0.20‰). Elemental analyses were conducted at the MNHN in Paris, France.

### Assessing the nitrogen fixation based on the natural abundance of ^15^N

The percentage of nitrogen derived from the atmosphere (%Ndfa) by leguminous species was calculated using the following formula (Shearer and Kohl 1986): %Ndfa = (δ^15^N_reference_ − δ^15^N_legume_)/(δ^15^N_reference_ − *β*) × 100, where *β* is the δ^15^N of the leguminous species growing on a N-free substrate (equal to 0 by default if the leguminous *β* is unknown). For *Vachellia* trees, four different %Ndfa values were calculated based on the δ^15^N values of the leaves, branches, wood and bark according to the respective reference species sampled near each *Vachellia* tree. The %Ndfa of other N_2_-fixing plants, such as *R. raetam* and *Vachellia* saplings, were calculated based on leaves. All reference species with δ^15^N values between −1‰ and +1‰ were removed, mainly because they introduced too many variations when using the %Ndfa calculation formula (a total of 12 out 171 δ^15^N values of reference species were omitted). Note that the %Ndfa values were also computed for the parasite (*P. acaciae*) because its N pool was hypothetically extracted from the leguminous host, and thus indirectly derived from the atmosphere by its host.

### *Vachellia* spp. tree characteristics

We performed a multi-level analysis of *Vachellia* trees concerning five different aspects: (1) identification of *Vachellia* species based on vernacular names, botanical description and molecular tools; (2) description of the tissue characteristics (wood, branches, bark or leaves); (3) description of the tree characteristics across ROIs; (4) distinction between saplings and mature trees; and (5) assessment of the presence/absence of the *P. acaciae* parasite.

### Morphological traits

The morphological traits of the 40 trees sampled in situ are reported in Table S1. They included the: (1) tree height (m), (2) trunk height (m), and (3) trunk diameter at breast height (DBH) (cm), and the plant health status. The health status of each tree was divided in three classes: healthy, parasitized, or grazed. One tree could have two different statuses (e.g. healthy-parasitized or grazed-parasitized). Regarding the analyses reported in Table S2, the healthy class included healthy trees and healthy-parasitized trees. The unhealthy class included parasitized trees, grazed trees and parasitized/grazed trees.

### *Vachellia* species identification

The first field investigation to identify the plant species focused on common plant names provided by local guides. Three vernacular/common names are given by local guides to identify each of the 40 *Vachellia* trees sampled (Table S1): طلح الحرّ—ṭalḥ l-ḥorr (=*taleh*), سيال—seyāl (=*seyal*), and صمر—ṣamr (=*samor*). All sampled *Vachellia* trees were annotated from VAC 01 to 40. All *taleh* individuals were located in ROIs 42, 43, 44, and 45 (from VAC 01 to VAC 20, with the exception of VAC 11). Both *seyal* and *samor* individuals were found in ROIs 46, 47, 48, and 49.

**Fig. 2:**
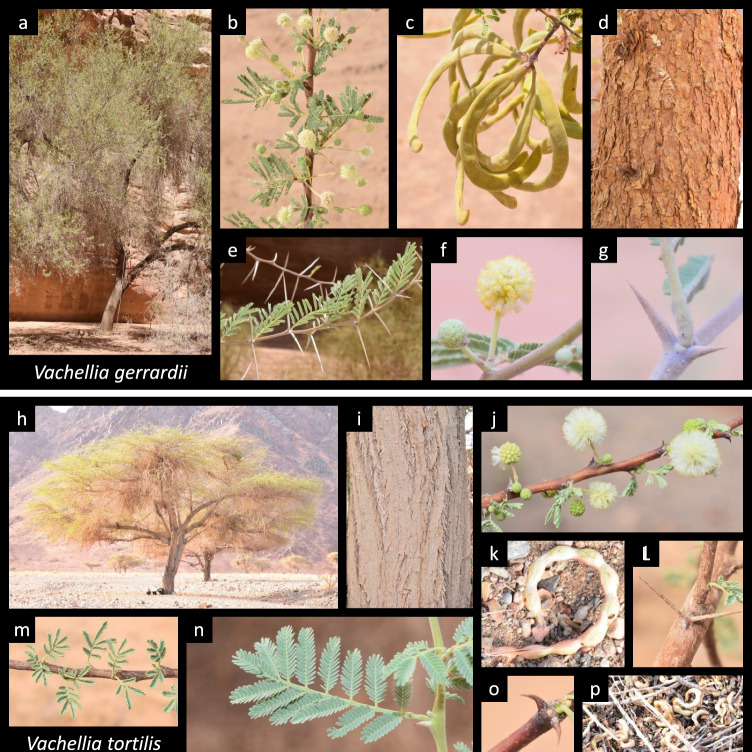
Morphological characteristics of *V. gerrardii* (**a**–**g**) and *V. tortilis* (**h**–**p**). *V. gerrardii* whole tree (**a**), branch with leaves and flowers (**b**), pods (**c**), trunk (**d**), branch with thorns (**e**), close-up of two flowers (**f**), close-up of thorns (**g**). *V. tortilis*: whole tree (**h**), trunk (**i**), branch with leaves and flowers (**j**), close-up of a pod (**k**), close-up of straight thorns (**l**), branch with leaves (**m**), close-up of a leaf (**n**), close-up of thorns (**o**), several pods found under one individual (**p**)

Based on the morphological traits (Fig. 2) and the morphological key published by Waly and Emad (2012), the *Vachellia* population in the AlUla region could host two potential species: *Vachellia gerrardii* (Benth.) P. J. H. Hurter, and *Vachellia tortilis* (Forssk.) Galasso & Banfi subsp. *raddiana* (Savi) Kyal. & Boatwr. (Kyalangalilwa et al. 2013; Mabberley 2017). The most useful criteria for differentiating the two species were mainly the pod color/shape and the type of stipule spine/thorn. For example, *Vachellia gerrardii* had a trunk color that varied from brown-reddish to black (Fig. 2d), with narrow falcate (sickle-shaped) pods (Fig. 2c) and straight thorns (Fig. 2e, g). In contrast, *Vachellia tortilis* had grey-brownish to blackish fissured bark (Fig. 2i), spiral pods with visible constriction (Fig. 2k, p), and both hooked and straight thorns (Fig. 2o, l). According to this botanical identification, 19 *Vachellia* trees from VAC 01 to 20 were identified as *V. gerrardii* (with the exception of VAC 11), whereas the remaining 21 trees were identified as *V. tortilis* (Table S1).

### *Vachellia* trees molecular identification

Sampled *Vachellia* spp. trees were identified using a single nuclear polymorphism method. DNA from 40 trees was extracted from silica-dried leaves using a protocol derived from Parker et al. (2005) in the ARCAD laboratories. Two chloroplast genes, i.e. *matK* and *rbcL*, were chosen because they are commonly used for plant identification (Lahaye et al. 2008), including *Acacia *sensu lato species (Ismail et al. 2020). The PCR program was as follows: 4 min initial denaturation at 94 °C; the first 12 cycles included 45 s denaturation at 94 °C, followed by 45 s denaturation at 65 °C with a temperature decrease of 0.7 °C per cycle and 90 s elongation at 72 °C; followed by 25 cycles of 45 s denaturation at 94 °C, 45 s hybridization at 65 °C, 90 s elongation at 72 °C; and a final hold of 5 min at 72 °C. Purification and bidirectional Sanger sequencing were performed by capillary electrophoresis on an ABI PRISM ® 3500 Genetic Analyzer. Newly obtained sequences were manually checked and corrected using MEGA v7.0.26. Contigs were assembled using the forward and reverse sequences. Reference sequences of *matK* and *rbcL* genes from other plant species were manually retrieved from the National Center for Biotechnology Information (NCBI), including two outgroups of *Senegalia* species and 20 other *Vachellia* species sequences (Table S3). All sequences were aligned online on the MultiAlin website (http://multalin.toulouse.inra.fr/multalin/). Once the sequences were aligned, the resulting FASTA file was copied and uploaded onto phylogeny.fr, a website dedicated to phylogenetic analysis. The phylogenetic trees generated were based on maximum likelihood with bootstrap values. The text on the phylogenetic trees was manually checked and edited using Inkscape software. Each *Vachellia* tree sampled was colored according to the vernacular and botanical identifications.

In parallel to phylogenetic trees, *Vachellia* spp. *matK* and *rbcL* gene sequences were analyzed with a nucleotide Basic Local Alignment Search Tool (BLAST) in the NCBI database. All *matK* and *rbcL* consensus sequences were thus submitted one by one for online BLAST analysis. The BLAST results for *matK* and *rbcL* are reported in Table S4 and S5, with the top three outputs, their accession numbers, and the percentage of similarity between the matching reference and our sequence.

### Statistical analyses

All statistical analyses were performed using R v4.3.1 (R Core Team 2022).

Principal component analyses were computed using raw data and the FactoMineR package (Lê et al. 2008) and visualized as biplots (combination of the correlations circle and the individuals graphic) using the factoextra package (Kassambara and Mundt 2020). Multiple correlations within a dataset were investigated through correlation matrix analyses and visualized using a network plot with the igraph package (Csárdi et al. 2023). A Spearman’s correlation matrix was first calculated. In parallel, the cor.mtest function from the corrplot package (Wei et al. 2021) computed the *P* values for each *ρ* value based on multiple non-parametric Spearman’s rank correlation tests. The multiple tests carried out and their associated *P* values were adjusted using the Bonferroni correction.

The network plot was computed using the graph_from_adjacency_matrix function in the igraph package. As this function only allows positive numbers, we used the absolute *ρ* values. A circle layout was chosen, where all vertices were equidistant to the center of the circle. Significant positive and negative correlations of the edges are shown in green and red with broader edges, respectively.

Wilcoxon–Mann–Whitney tests were used to test the distribution independence. Kruskal–Wallis tests, followed by post hoc Dunn tests were used for multiple comparison of medians. Significant different distributions (at *P* < 0.05) are indicated with letters (‘a’, ‘b’, etc.). Treatments with the same letters are not significantly different (e.g. the level ‘ab’ is not significantly different from the level ‘a’ or ‘b’).

In order to assess the stability of the data dispersion per group, coefficients of variation (CV) were calculated with the following formula: CV = standard deviation/mean × 100, and expressed as percentages.

## Results

### All plant characteristics in relation to their biology

**Fig. 3:**
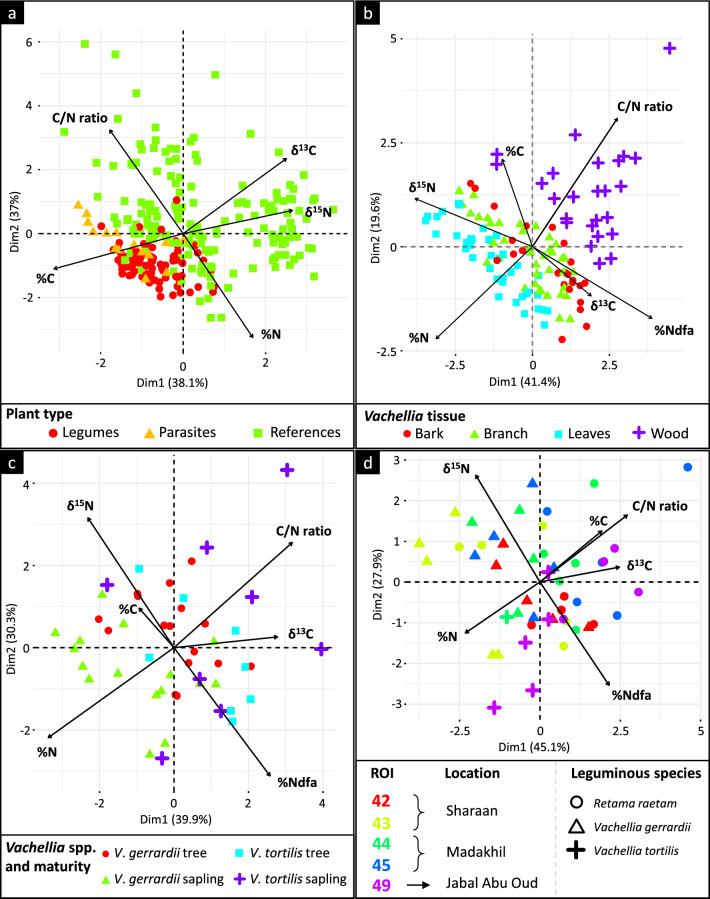
Principal component analyses (PCA) of desert plants sampled in the AlUla region. Overview of all plants collected in relation to their biological type: legumes, parasite or reference species (**a**). Analysis of the characteristics of *Vachellia* tissues (**b**). Comparison of mature trees and saplings between *V. gerrardii* and *V. tortilis* (**c**). Analysis of the characteristics of *R. raetam* co-occurring with *Vachellia* spp. (**d**)

All plant data (leguminous species + parasites + references species) collected in the AlUla region were compared to each other based on their N and C contents, isotopic signatures, and C/N ratios (Fig. 3a; Table 2a). Data were grouped according to the biological plant type considered for the study: (1) leguminous species (*Vachellia* spp. and *R. raetam*), (2) parasite species (*P. acaciae*), and (3) non-N_2_-fixing reference species harvested in the vicinity of *Vachellia* trees. In Fig. 3a, dimensions 1 and 2 respectively explain 38.1 and 37.0% of the total variation. The three groups presented different characteristics: the reference species had significantly higher N and C isotopic signatures and lower C content than leguminous and parasite species (Table 2a). Leguminous species showed the highest N content (2.4 ± 0.4%) compared to the parasite (1.7 ± 0.5%) and other reference species (2.0 ± 0.9%). The additional correlations are presented in Table S6. In Table S7, the detailed values of every species sampled showed that *Vachellia* spp. and *R. raetam* had scant N content variations (CVs of 12% and 13%, respectively) compared to other species, such as *C. colocynthis*, *E. aphylla*, *L. shawii*, *O. baccatus*, *P. turgidum*, *S. baryosma*, and *S. italica*, which had CVs > 30%, all ROIs combined. The C content was more stable than the N content, with CVs < 9%, all species considered.

**Table 2:** Measures of the N and C contents, C/N ratio, isotopic signatures of δ^15^N and δ^13^C, and %Ndfa for plants sampled in the AlUla region

Type of analysis	Groups	*n*	N content (%)	C content (%)	C/N ratio	δ^15^N (‰)	δ^13^C (‰)	%Ndfa
(a) Plant type	Legume	87	2.43 ± 0.44 a	47.2 ± 2.5 a	23.4 ± 5.0 a	2.58 ± 2.48 b	−27.7 ± 1.4 b	NA
	Parasite	14	1.66 ± 0.46 b	46.3 ± 1.7 a	35.0 ± 10.6 b	2.01 ± 2.74 b	−30.1 ± 1.4 b	NA
	Reference	149	2.00 ± 0.93 b	40.9 ± 5.3 b	32.5 ± 24.4 ab	5.26 ± 2.65 a	−20.8 ± 6.7 a	NA
(b) Tissues of *Vachellia*	Bark	24	0.90 ± 0.33 c	43.3 ± 1.9 c	62 ± 19 b	1.16 ± 2.52 ab	−27.5 ± 1.0 a	74 ± 35 ab
	Branch	40	1.33 ± 0.31 b	45.3 ± 1.9 b	42 ± 10 b	1.45 ± 2.28 a	−27.8 ± 1.1 a	69 ± 35 ab
	Leaves	40	2.38 ± 0.30 a	46.4 ± 1.6 ab	23 ± 3 b	2.64 ± 2.32 a	−28.0 ± 1.1 a	54 ± 34 b
	Wood	25	0.22 ± 0.12 d	46.2 ± 2.3 a	327 ± 238 a	−0.12 ± 2.42 b	−27.3 ± 1.0 a	85 ± 29 a
(c) Maturity of *Vachellia*	*V. gerrardii* tree	16	2.34 ± 0.30 b	46.8 ± 1.4 a	23.8 ± 3.3 a	2.93 ± 1.90 a	−28.7 ± 0.6 b	37 ± 36 a
	*V. gerrardii* sapling	16	2.94 ± 0.42 a	45.9 ± 0.9 ab	18.7 ± 3.4 b	3.29 ± 3.01 a	−28.8 ± 0.8 b	41 ± 40 a
	*V. tortilis* tree	8	2.30 ± 0.33 b	45.1 ± 1.6 ab	23.2 ± 2.8 ab	2.81 ± 3.02 a	−27.5 ± 0.8 a	63 ± 38 a
	*V. tortilis* sapling	8	2.18 ± 0.72 b	44.3 ± 1.8 b	26.1 ± 9.0 a	3.83 ± 3.60 a	−27.1 ± 1.3 a	47 ± 45 a
(d) *Retama* & *Vachellia*	*R. raetam*	23	2.25 ± 0.30 a	50.3 ± 1.5 a	26.6 ± 4.2 a	1.55 ± 1.41 ab	−26.6 ± 1.5 a	65 ± 30 a
	*V. gerrardii*	19	2.33 ± 0.28 a	47.0 ± 1.4 b	23.8 ± 3.1 a	2.92 ± 1.92 a	−28.7 ± 0.6 b	39 ± 34 b
	*V. tortilis*	6	2.34 ± 0.38 a	44.6 ± 2.1 c	22.7 ± 3.4 a	0.9 ± 1.08 b	−27.9 ± 0.9 ab	80 ± 18 a

Four types of analyses are included: (a) Analysis of all plants in relation to their biological type (legume, parasite, or reference species). (b) Comparison of the characteristics of tissues sampled from *Vachellia* trees. (c) Analysis of differences between mature trees and saplings among *Vachellia* species. (d) Comparisons between *R. raetam* characteristics and co-occurring *Vachellia* species. Each type of analysis is subdivided into groups, with the number of replicates (*n*) indicated. Results are given as means ± standard deviations. Significantly different groups (*P* < 0.05) are symbolized with letters

### *Vachellia* spp. characteristics

#### *Vachellia* species identification

Three common names were found to be used to identify *Vachellia* trees in the AlUla region. However, only two species were considered in the present study based on the botanical identification: *V. gerrardii* and *V. tortilis* (Fig. 2). Interestingly, all *taleh* individuals matched with *V. gerrardii*, whereas all *samor* individuals corresponded to *V. tortilis*. Regarding *seyal* individuals, one of them was classified as *V. gerrardii*, and 11 others as *V. tortilis* (Table S1; Figs. S3 and S4).

Regarding the molecular identification, phylogenetic trees identified based on *matK* and *rbcL* (Figs. S3, S4) showed that the *Vachellia* species sampled (VAC 01 to VAC 40) were very close to each other on both phylogenetic trees, thus providing little information for the identification of *Vachellia* species. However, the information provided by *matK* was different from that provided by *rbcL*. Indeed, two *Vachellia* clusters emerged with the *matK* gene (VAC 01 to VAC 20 versus VAC 21 to VAC 40), whereas only one group was present based on *rbcL* sequence information. The BLAST results were convergent with the phylogenetic findings: the *matK* sequences of the first 20 trees (VAC 01 to VAC 20) were similar to those of several leguminous species, including *Acacia iraqensis*, *Vachellia gerrardii* var. *gerrardii*, and *Vachellia gerrardii* var. *najdensis* species (Table S4). The following 20 *matK* sequences of *Vachellia* trees (with the addition of VAC 11) were similar to *Vachellia tortilis* subsp. *raddiana*, *Vachellia tortilis* subsp. *tortilis*, and *Vachellia tortilis* isolate UHURU1133-14. No sequence was generated for VAC 16. The top three BLAST results for all *rbcL* sequences (VAC 01 to VAC 40) gave exactly the same outputs: *Vachellia tortilis* subsp. *raddiana* (two times), and *Senegalia senegal* (L.) Britton (Table S5).

According to all of the elements mentioned, we considered two *Vachellia* species for this study, i.e. *Vachellia gerrardii* (VAC 01 to VAC 20, with the exception of VAC 11), and *Vachellia tortilis* (VAC 21 to VAC 40, including VAC 11).

#### *Vachellia* spp. characteristics in relation to the sampled tissue

The principal component analysis (PCA) results presented in Fig. 3b explained 61% of the total variation. The different *Vachellia* spp. tissues were spatially separated on both dimensions 1 and 2, indicating different tissue characteristics and separation according to the N and C contents, C/N ratio, δ^15^N signature, and associated %Ndfa. The numerical details of the analysis are presented in Table 2b. Leaves had the highest N content (2.38 ± 0.30%) compared to other tissues, whereas wood had the lowest N content (0.22 ± 0.12%). Moreover, the δ^15^N signature of *Vachellia* leaves (2.64 ± 2.32‰) was significantly higher than that of wood tissues, i.e. close to 0 ‰ (−0.12 ± 2.42‰). The BNF calculated for leaf tissues was thus significantly lower (54 ± 34%Ndfa) compared to that of wood tissues (85 ± 29%Ndfa). These results were supported by the correlation matrix presented in Table S8, where the calculated %Ndfa was significantly and negatively correlated with δ^15^N (*ρ* = −0.94; *P* < 0.001).

#### *Vachellia* characteristics across ROIs

*Vachellia* characteristics across ROIs are shown in Fig. S5, and are presented in Table 2b. The N contents of *Vachellia* trees among ROIs were found to be not significantly different and ranged from 2.10 to 2.63% (Table 3a), while some differences in C content were detected between ROIs, but without influencing the average plant C/N ratio. Two levels of isotopic signatures of both N and C were found between the ROIs (Table 3b). The %Ndfa was highly variable across ROIs (28%Ndfa on average in ROIs 43 and 48 versus 82 and 83%Ndfa in ROIs 49 and 46, respectively). However, statistical analysis of these variations showed that they were not significantly different, mainly because of the high intraspecific variability between trees growing in the same ROI. The morphological traits reported in Table 3c showed that smaller trees were growing in ROIs 46 (5.6 ± 0.7 m) and ROI 49 (5.2 ± 1.4 m) compared to ROI 43 (10.2 ± 3.0 m), with no variation in trunk height or DBH across ROIs. The average tree heights of *V. gerrardii* (8.19 ± 2.34 m) and *V. tortilis* (6.61 ± 1.77 m) were significantly different. The correlation matrix based on the tree characteristics is presented in Table 3b.

**Table 3:** Multiple means comparisons of *Vachellia* characteristics: N and C contents, with C/N ratio (a), isotopic signatures and %Ndfa (b), and morphological traits: tree and trunk heights, with trunk diameter at breast height (c)

(a) Nitrogen and carbon contents with C/N ratio			
*Vachellia* ROI	Nitrogen (%)	Carbon (%)	C/N ratio
ROI 42	2.35 ± 0.20 a	46.7 ± 0.6 ab	24.3 ± 2.6 a
ROI 43	2.19 ± 0.18 a	45.7 ± 1.0 bc	26.3 ± 8.8 a
ROI 44	2.60 ± 0.25 a	48.4 ± 0.7 a	25.1 ± 3.4 a
ROI 45	2.38 ± 0.37 a	47.4 ± 1.7 ab	20.5 ± 2.0 a
ROI 46	2.63 ± 0.29 a	46.1 ± 1.3 ab	23.3 ± 1.8 a
ROI 47	2.35 ± 0.24 a	46.5 ± 1.2 ab	20.8 ± 1.7 a
ROI 48	2.23 ± 0.26 a	46.7 ± 1.1 ab	23.4 ± 4.3 a
ROI 49	2.10 ± 0.58 a	43.7 ± 1.1 c	25.0 ± 2.1 a

(b) Nitrogen and carbon isotopic signatures and %Ndfa			
*Vachellia* ROI	δ^15^N (‰)	δ^13^C (‰)	%Ndfa
ROI 42	1.45 ± 1.30 b	−28.2 ± 0.51 ab	52 ± 42 a
ROI 43	3.63 ± 2.59 ab	−29.3 ± 0.59 b	28 ± 39 a
ROI 44	2.98 ± 1.39 b	−29.0 ± 0.35 b	41 ± 29 a
ROI 45	3.45 ± 1.69 ab	−28.2 ± 0.44 ab	40 ± 29 a
ROI 46	0.92 ± 1.57 b	−27.1 ± 0.60 a	83 ± 26 a
ROI 47	1.80 ± 1.68 b	−27.8 ± 1.20 ab	75 ± 24 a
ROI 48	6.39 ± 1.58 a	−26.9 ± 1.12 a	28 ± 17 a
ROI 49	0.67 ± 1.03 b	−27.8 ± 0.93 ab	82 ± 19 a

(c) Morphological traits of *Vachellia* trees			
*Vachellia* ROI	Tree height (m)	Trunk height (m)	Trunk DBH (cm)
ROI 42	7.9 ± 1.4 ab	3.01 ± 0.67 a	120 ± 39 a
ROI 43	10.2 ± 3.0 a	3.16 ± 0.23 a	110 ± 15 a
ROI 44	8.2 ± 2.1 ab	3.40 ± 1.14 a	128 ± 45 a
ROI 45	6.9 ± 1.8 ab	2.30 ± 0.59 a	136 ± 34 a
ROI 46	5.6 ± 0.7 b	2.18 ± 0.25 a	126 ± 99 a
ROI 47	8.1 ± 2.3 ab	2.75 ± 0.99 a	153 ± 95 a
ROI 48	7.4 ± 1.0 ab	2.70 ± 0.84 a	224 ± 34 a
ROI 49	5.2 ± 1.4 b	1.90 ± 0.82 a	104 ± 37 a

Results are given as means ± standard deviations. Kruskal–Wallis tests followed by Dunn tests were used for multiple means comparisons. Significantly different means (at *P* < 0.05) are indicated with letters (‘a’, ‘b’, etc.). Treatments with the same letters are not significantly different (e.g. the ‘ab’ level is not significantly different from the ‘a’ or ‘b’ levels)

An additional analysis of the tree health status is presented in Table S2. Among the 40 trees sampled, we discriminated healthy trees from unhealthy trees (Table S1 for further details). Land use significantly affected the proportion of healthy trees in the natural reserve (16/20 healthy trees), archaeological site (5/10 healthy trees) and public domain (0/10 healthy trees) (Table S2a). No relationship was found between the health status and morphological plant traits (Table S2b). However, the *V. gerrardii* population was significantly healthier (15 healthy trees out of 19 trees overall) than the *V. tortilis* population (6 healthy trees out of 21 trees overall) (Table S2c).

#### *Vachellia* saplings versus mature trees

A comparison of *V. gerrardii* and *V. tortilis* trees and their associated saplings is shown in Fig. 3c and Table 2c. The leaf N content of *V. gerrardii* saplings (2.94 ± 0.42%) was significantly higher compared to that of *V. tortilis* mature trees (2.30 ± 0.33%), *V. gerrardii* trees (2.34 ± 0.30%) and saplings (2.18 ± 0.72%). The C content of *V. gerrardii* trees (46.8 ± 1.4%) was significantly higher compared to that of *V. tortilis* saplings (44.3 ± 1.8%). Regarding the δ^15^N and %Ndfa in *V. tortilis* and *V. gerrardii* saplings and mature trees, no statistical differences were found, mainly because of the high variations in these parameters. However, the δ^13^C signatures differed between species but not between saplings and mature trees. Additional correlations are presented in Table S10.

#### The parasite–host relationship between *Plicosepalus acaciae*, the witch broom disease and *Vachellia* trees

**Fig. 4:**
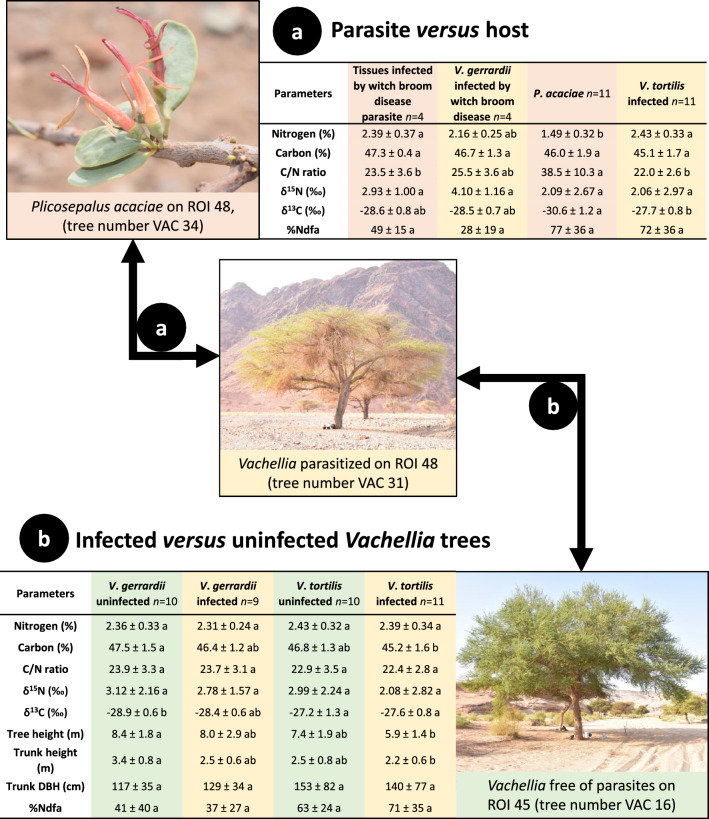
Analyses of the parasite:host relationship between *Plicosepalus acaciae* and the witch broom disease (WBD) with *Vachellia* trees. The two sections present the C and N contents and isotopic signatures of the two parasites and their respective *Vachellia* hosts (**a**), and the C and N contents, isotopic signatures, growth, and nitrogen fixation differences between parasitized and non-parasitized *Vachellia* trees according to the species (**b**). Values are expressed as mean ± standard deviation (10 *P. acaciae* samples, 4 WBD samples). Kruskal–Wallis tests followed by Dunn tests were used for multiple means comparisons. Significantly different means (at *P* < 0.05) are indicated with letters (‘a’, ‘b’, etc.). Treatments with the same letters are not significantly different (e.g. the ‘ab’ level is not significantly different from the ‘a’ or ‘b’ levels)

**Fig. 5:**
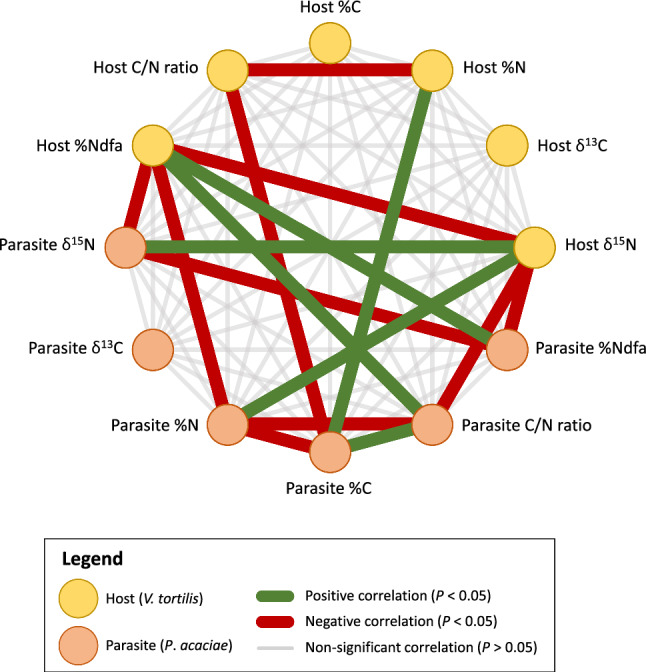
Network correlation plot of the *P. acaciae* parasite and its *V. tortilis* host. Parameters measured include element contents and isotopic signatures of carbon and nitrogen, C/N ratio and the %Ndfa of both species. Significant positive and negative correlations (at *P* < 0.05) are highlighted in green and red, respectively

A total of 10 *P. acaciae* samples were obtained on *V. tortilis* trees in ROIs 46, 47, 48, and 49, whereas a total of four WBD infected tissues were sampled exclusively in Madakhil, from ROIs 45 (1 individual) and 46 from *V. gerrardii* (3 individuals; Table 1). The parasitism patterns of *Vachellia* trees are presented in Fig. 4 and were investigated on the basis of: (1) the characteristics of the WBD parasitized tissues and *P. acaciae* compared to their respective hosts (Fig. 4a), (2) the correlation between the parasite and its host (Fig. 5; Table S12), and (3) the differences between infected and uninfected *Vachellia* tree populations, and also in relation to *Vachellia* species (Fig. 4b). Among all the species sampled, *P. acaciae* had the lowest δ^13^C values, with an average of −30.6‰ (Table S7).

#### Comparison of *P. acaciae* and infected *Vachellia* trees

The N and C contents, isotope, and BNF features in the host and its parasite were investigated (Fig. 4a). The average N content of the *P. acaciae* parasite growing on *V. tortilis* trees (1.49 ± 0.32%) was significantly lower than that of its host (2.43 ± 0.33%). However, the N content in WBD infected tissues was similar to that of uninfected tissues in *V. gerrardii* trees: 2.39 ± 0.37% and 2.16 ± 0.25%, respectively. The C content was similar among all the biological materials. In *V. tortilis*, the C/N ratio differed between *P. acaciae* (37.8 ± 10.1) and its host (21.9 ± 2.5), which was not the case for WBD infected tissues compared to uninfected tissues in *V. gerrardii*. The δ^15^N signatures and the resulting %Ndfa computed between infected and uninfected trees were similar, with high variability between and within sites (Fig. 4a). Correlations between the parasite and its host (*V. gerrardii* and *V. tortilis* combined) are presented in Fig. 5. Out of the 60 correlations tested overall, 16 were significant (*P* < 0.05), including 10 negative and 6 positive correlations. Six of them were intraspecific (i.e. the host δ^15^N with the host %Ndfa, or the parasite C content with the parasite C/N ratio) and 10 of them were interspecific (i.e. the host δ^15^N with the parasite δ^15^N, or the parasite C content with the host N content). The parasite δ^13^C, host δ^13^C and host C contents were not correlated with the other parameters.

#### Comparison of infected and uninfected *Vachellia* trees

Further investigations were carried out on parasitism in infected and uninfected *V. gerrardii* and *V. tortilis* populations (Fig. 5b). Most of the parameters measured (N content, C/N ratio, δ^15^N signature, trunk DBH and %Ndfa) were similar among the populations, regardless of the presence of the parasite or the *Vachellia* species considered. Regarding the tree height, trunk height and C content, infected *V. tortilis* trees had significantly lower values than those of uninfected *V. gerrardii* trees. The two *Vachellia* populations had a different parasitic status. The only parameter influenced by the plant species was the δ^13^C of *V. gerrardii* and *V. tortilis* uninfected trees (−28.9 ± 0.6‰ and −27.0 ± 0.9‰, respectively).

### Characteristics and BNF of *R. raetam* versus *V. gerrardii* and *V. tortilis*

A total of 19 *R. raetam* individuals were sampled (Table 1) and found to co-occur with *Vachellia* trees in both Sharaan (ROIs 42 and 43) and Madakhil (ROIs 44 and 45) Nature Reserves, and in the Jabal Abu Oud open public domain (ROI 49). First, the *R. raetam* population was investigated across ROIs and then compared to other leguminous species, i.e. *V. gerrardii* and *V. tortilis*.

#### Intraspecific analysis of *R. raetam* across locations

Plant parameters, such as N content, C/N ratio, δ^15^N, and %Ndfa, were stable across the different locations (Fig. S6; Table S12). Only the C content and isotopic signature differed between Madakhil and Sharaan. Regarding the BNF, the average %Ndfa of *R. raetam* reached 80% in Jabal Abu Oud, 64% in Madakhil and 60% in Sharaan.

#### Comparison of *R. raetam* to *V. gerrardii* and *V. tortilis*

*Retama raetam* characteristics compared to those of co-occurring *Vachellia* trees are shown in Fig. 3d and are analyzed in Table 2d. *Retama raetam* showed the highest C content, C/N ratio and δ^13^C signature compared to *Vachellia* species. The overall BNF of *R. raetam* (65.2%) was significantly higher than that of *V. gerrardii* (39.1%) and similar to that of *V. tortilis* (79.5%), whereas the N content was stable among the three leguminous species (Table 2d). Additional correlations are presented in Table S13.

## Discussion

### Empirical, molecular, and botanical identification of *Vachellia* trees

Although the common names given to the *Vachellia* trees found in the AlUla region were diverse, they were generally in line with the botanical identifications, where *taleh* and *samor* were affiliated with *V. gerrardii* and *V. tortilis*, while the identification of *seyal* individuals was more confusing (Table S1). The main confusion may have been due to the three words *taleh*, *samor* and *seyal*, because all of them are commonly used to describe *Acacia raddiana Savi* (*Vachellia tortilis*) in Africa and the Middle East (Bellakhdar 1997). Regarding the molecular data, *matK* and *rbcL* are quite interesting markers to discriminate some *Acacia* species and even to develop unique species barcodes (Ismail et al. 2020). In our study, molecular identification of *Vachellia* trees based on *matK* and *rbcL* markers was sufficient for genus level identification, but was not accurate enough to differentiate *V. gerrardii* from *V. tortilis*, which was also noted in a large-scale molecular identification study on *Acacia* species present in Saudi Arabia (Abdel-Hamid et al. 2021). This poor molecular interspecific divergence may have been due to potential hybridizations and different ploidy levels, as often reported in *Acacia *sensu lato species (Blakesley et al. 2002; Odee et al. 2015). Nonetheless, the dichotomous key used to identify *Vachellia* trees (Waly and Emad 2012), based on morphological traits of organs, such as thorns and pods, remains the most accurate identification method to distinguish *Vachellia* species regardless of their genetic characteristics.

### Steadiness of the growth and ecological aspects of *Vachellia* spp. and *Retama raetam* populations in hyper-arid natural ecosystems

Although differences in the health status of *Vachellia* trees were observed in the field (Fig. S1e, f) and may have been related to the parasite prevalence and/or anthropic pressure through land use and grazing, one of the major results of this study concerned the steadiness of the growth and ecological aspects of desert tree/shrubby legumes, such as *V. gerrardii*, *V. tortilis*, and *R. raetam*, in the eight ROIs, where the BNF was variable (Tables S9, S11). These results are in line with those reported by Schulze et al. (1991) along an aridity gradient for several *Acacia *sensu lato species in Namibia.

*Vachellia gerrardii* had a lower BNF than *V. tortilis* in the field, and both maintained high, steady and similar inner N contents. Except for ROI 48, the BNF of *V. tortilis* (83, 75, and 82% on ROIs 46, 47, and 49, respectively) was higher than that reported by Ndoye et al. (1995) in *Acacia raddiana* in Senegal (62%, using the ^15^N dilution isotope method at a young development stage). The low BNF assessed on ROI 48 (Wady Al Ward) may have been due to the extent of domestic livestock herd grazing in the area (Fig. S1g, h). Only *Vachellia* trees and *Haloxylon salicornicum* were sampled at this site, thus highlighting the intense grazing pressure in the area. The presence of abundant local fauna leads to substantial urine and feces deposition and, when the animals seek cover and shade under *Vachellia* trees (Fig. S1i), their excretions accumulate around the tree base. In the long run, regular urine introduction may increase the δ^15^N of the soil and be assimilated by trees (Tonn et al. 2019), thus increasing their δ^15^N content (6.39‰ for *Vachellia* trees growing in ROI 48). As a result, the BNF of *V. tortilis* in ROI 48 may have been underestimated and/or inhibited by the presence of camel, goat and sheep herds and donkeys. Another parameter that could potentially impact BNF estimation is the choice of tissue used for calculating the %Ndfa. Although leaves are a rare resource in desert environments, they are also the best tissue to assess the BNF of *Vachellia* trees because they have the highest N content (2.4%) compared to woody tissues (0.2% for wood; 0.9% for bark; 1.3% for branches). Assessing the ^15^N natural abundance in a desert environment is challenging, mainly because vegetation is scarce, and common reference species patterns must be found in situ between study sites. However, in our study we successfully assessed the in situ BNF of *V. gerrardii* and *V. tortilis* populations using 12 local reference species, thereby highlighting that studying plant–microbe mutualistic symbioses in hyper-arid environments is both feasible and useful, thus encouraging more field studies in harsh environments.

*Retama raetam* (also known as retem or white broom) is a desert shrubby legume with high multipurpose potential for its medicinal/pharmacological properties and its effectiveness for the ecological restoration of degraded lands (Al-Sharari et al. 2020). One study assessed the in situ BNF of *R. raetam* in the Negev Desert and found an average of 74% BNF, with variations among sites ranging from 46 to 86% (Russow et al. 2004). Our study also confirmed that the BNF of retem was highly efficient in natural ecosystems (64% in Madakhil, and 60% in Sharaan Nature Reserves, and 80% in Jabal Abu Oud), and the plant showed little variation among locations (Fig. 3d; Table S12). Retem is highly adapted to dry and arid conditions, with its deep root system, the ability to limit evapotranspiration through leaf shedding, and its slim and photosynthetic branches (León-González et al. 2018). The introduction and development of retem in nature reserves, such as Sharaan and Madakhil, is promising for the preservation of local fauna because this N_2_-fixing shrub meets the requirements to be a substitute for conventional foraging species (Barakat et al. 2013). However, retem shrubs are an easy target for wandering herbivorous animal species, and may not withstand intensive grazing in certain areas where local fauna is abundant and uncontrolled.

### Parasitism and ecological aspects of *Vachellia* trees

The parasite:host N ratio is a useful variable for gaining further insight into the extent of mistletoe parasitism. Mistletoes have been suggested as potential sinks capable of accumulating N from their hosts (Panvini and Eickmeier 1993). In our study, the average leaf N contents of *P. acaciae* and *Vachellia* were 1.49 and 2.43%, respectively (Fig. 4a), resulting in a parasite:host N ratio of 0.60. In other species, the interaction between mistletoe, *Phoradendron leucarpum* (Raf.) Reveal & M.C. Johnst., and 46 different hosts reported parasite:host N ratios ranging from 0.97 to 2.88 (Panvini and Eickmeier 1993). However, a similar study on *P. acaciae* growing on *Vachellia* trees in the Negev Desert reported a parasite:host N ratio similar to ours, i.e. 0.65 (Bowie and Ward 2004). We hypothesized that the physiology/metabolism of mistletoe is specific to its environment and accumulates less N in hyper-arid conditions, which may alleviate their impact on their host and enhance plant fitness. *P. acaciae* growing on *Vachellia* trees bear leaves and are thus able to benefit from their own photosynthesis (Fig. 1d). The main inputs for this reaction are carbon dioxide and water. Aerial mistletoes have no access to underground water, so they need to extract water from their host, and their water use efficiency is lower than that of their host (Ehleringer et al. 1986). Poor water use efficiency can be detected through low C isotopic signatures (δ^13^C) (Bchir et al. 2016). In our study, we found a significant difference in δ^13^C between the parasite (−30.1‰) and the host (−27.7‰), suggesting that the parasite had a poorer water use efficiency compared to that of the host (and the lowest δ^13^C of all plants sampled in the study; Table S7). Moreover, *V. tortilis* growing in the hyper-arid Negev Desert is known to have limited stomatal closure control, throughout the year and even under a wide range of relative humidity conditions ranging from 4 to 94% (Do et al. 2008; Winters et al. 2018). Links between the δ^13^C and the BNF of legume trees have also been demonstrated in Namibia in several *Acacia *sensu lato species (Schulze et al. 1991). Regarding the witch broom disease, the nature and origin of the causal agent infecting *Vachellia* trees in Saudi Arabia is still unknown, and should be carefully investigated first. Moreover, monitoring the spread of the WBD is crucial to avoid an outbreak, potentially causing minor to extensive damages to crop cultures according to the causal agent. In theory, infected trees hosts are forced to feed unwanted plant tissues, thus wasting a part of their water and nutrient resources. However, these deformities resulting from the WBD are not systemically detrimental, and can also be beneficial and create a better habitat for other organisms (Pires et al. 2020). Such relationships are complex to decipher, and emphasize the trickiness of understanding the respective roles of organisms involved in these interactions.

Based on these results, parasitized *Vachellia* trees infected by *P. acaciae* and/or WBD could be expected to suffer from greater drought stress compared to uninfected trees. However, our field data revealed no difference in δ^13^C between parasitized and uninfected *Vachellia* trees (Fig. 4b), suggesting that the parasite had no noteworthy impact on its host’s water management. One possible explanation could be that *V. tortilis* and *V. gerrardii* trees in the AlUla region have developed deep root systems, similar to those of *V. tortilis* growing in Kenya (Belsky 1994), thus providing permanent access to deep groundwater. As long as groundwater is replenished (i.e., via annual rainfall), *Vachellia* trees commonly infected by *P. acaciae* or WBD could potentially continue growing in hyper-arid environments (Winters et al. 2018). However, three elements may threaten the conservation of these natural ecosystems: (1) several studies have reported that groundwater and aquifer recharge rates in anthropized desert environments are insufficient to fully replenish them (Seraphin et al. 2022), (2) Saudi Arabia is warming through climate change, with an increase in extreme temperatures (Almazroui et al. 2014), and (3) the increasing demography of the country, resulting in a higher demand for freshwater (Chowdhury and Al-Zahrani 2015). In the light of all of these combined elements, maintaining the unique biodiversity and beauty of the local nature reserves and ecosystems may be challenging in the near future as climate change will inevitably amplify the scarcity of water, a resource that is already jeopardized in hyper-arid ecosystems.

## Conclusion

*Vachellia* spp. trees, confronting both anthropic pressures and parasitism within hyper-arid ecosystems, developed multifarious ecological traits and established mutualistic relationships. The steadiness of N inner contents of both *Vachellia* spp. and *R. raetam* compared to co-occurring non-leguminous species is the strong indicator that BNF genuinely contributes to plant’s fitness. Although *Vachellia* spp. trees are often infected by mistletoes and WBD, no detrimental effect was detected on plant growth, and C and N contents, highlighting once more the resilience of *Vachellia* species. Overgrazing may be harmful to wild plants, but recent fencing policies and moderate control over local fauna showed rapid improvement of the natural ecosystems. Nevertheless, safeguarding the interconnectedness and intrinsic value of these ecosystems necessitates a vigilant focus on natural water resources and their management. The investigation of these species significantly deepened our comprehension of their pivotal role in hyper-arid ecosystems, offering potential applications in land restoration efforts, particularly within the context of climate change. One step towards successful land restoration has already been taken because *Vachellia* trees are already acknowledge by locals and is a useful resource in their daily life (forage, medicinal properties, etc.). In conclusion, *Vachellia* trees are emblematic trees for both interconnectedness of hyper-arid ecosystems and for people where land restoration and climate change are major topics.

### Supplementary Information

Below is the link to the electronic supplementary material.

Supplementary file1 (PDF 563 KB)

Supplementary file2 (PDF 660 KB)

Supplementary file3 (PDF 222 KB)

Supplementary file4 (PDF 269 KB)

Supplementary file5 (PDF 156 KB)

Supplementary file6 (PDF 91 KB)

Supplementary file7 (DOCX 13 KB)

Supplementary file8 (DOCX 22 KB)

Supplementary file9 (DOCX 14 KB)

Supplementary file10 (DOCX 15 KB)

Supplementary file11 (DOCX 17 KB)

Supplementary file12 (DOCX 17 KB)

Supplementary file13 (DOCX 14 KB)

Supplementary file14 (DOCX 20 KB)

Supplementary file15 (DOCX 14 KB)

Supplementary file16 (DOCX 16 KB)

Supplementary file17 (DOCX 14 KB)

Supplementary file18 (DOCX 17 KB)

Supplementary file19 (DOCX 14 KB)

Supplementary file20 (DOCX 14 KB)

## Data Availability

All the data supporting the findings of this study are included in this article and in Supplementary material. Further inquiries can be directed to the corresponding author.

## Abbreviations

%Ndfa: Percentage of nitrogen derived from the atmosphere
BNF: Biological nitrogen fixation
DBH: Trunk diameter at breast height
ROI: Region of interest
VAC: Abbreviation of *Vachellia* used for sample numbering
WBD: Witch broom disease
