# Correction to: Ecological aspects and relationships of the emblematic *Vachellia* spp. exposed to anthropic pressures and parasitism in natural hyper-arid ecosystems: ethnobotanical elements, morphology, and biological nitrogen fixation

**DOI:** 10.1007/s00425-024-04436-9

**Published:** 2024-05-15

**Authors:** Bryan Vincent, Julie Bourillon, Karine Gotty, Hassan Boukcim, Marc-André Selosse, Aurélie Cambou, Coraline Damasio, Mathis Voisin, Stéphane Boivin, Tomas Figura, Jérôme Nespoulous, Antoine Galiana, Kenji Maurice, Marc Ducousso

**Affiliations:** 1 CIRAD, UMR113 LSTM, TA A-82⁄J, Campus International de Baillarguet, 34398 Montpellier Cedex 5, France; 2 Department of Research and Development, VALORHIZ, 1900, Boulevard de la Lironde, PSIII, Parc Scientifique Agropolis, 34980 Montferrier sur Lez, France; 3 Institut Systématique Evolution Biodiversité (ISYEB), Muséum National d’Histoire Naturelle (MNHN), CNRS, Sorbonne Université, EPHE, 57 rue Cuvier, CP39, 75005 Paris, France; 4 Department of Plant Taxonomy and Nature Conservation, University of Gdańsk, Wita Stwosza 59, 80-308 Gdańsk, Poland; 5 Institut Universitaire de France, Paris, France; 6 Eco&Sols, IRD, Université de Montpellier, CIRAD, INRAE, Institut Agro, Montpellier, France; 7 Department of Mycorrhizal Symbioses, Institute of Botany, Czech Academy of Sciences, Lesní 322, Průhonice, Czech Republic; 8 Understanding Evolution Group, Naturalis Biodiversity Center, Darwinweg 2, 2333 CR, Leiden, The Netherlands

**Correction to: Planta (2024) 259:132** 10.1007/s00425-024-04407-0

In this article, the text “at AI” was incorrectly replaced with “et al.” under the Abstract section. The complete sentence is given below.

In this region, we investigated the characteristics of desert legumes in two nature reserves (Sharaan and Madakhil), at one archaeological site (Hegra), and in open public domains at AI Ward and Jabal Abu Oud. Biological nitrogen fixation (BNF), isotopes and N and C contents were investigated through multiple lenses, including parasitism, plant tissues, species identification, plant maturity, health status and plant growth.

The original article has been corrected.

