# Supplementary figures and images for: Ecological aspects and relationships of the emblematic Vachellia spp. exposed to anthropic pressures and parasitism in natural hyper-arid ecosystems: ethnobotanical elements, morphology, and biological nitrogen fixation
Source: Planta. 2024 Apr 25;259(6):132. doi: 10.1007/s00425-024-04407-0 (PMC11045644; doi:10.1007/s00425-024-04407-0)

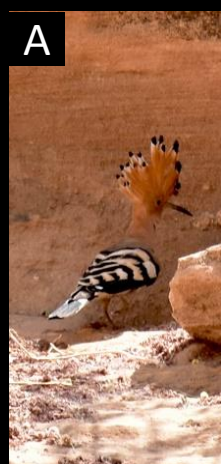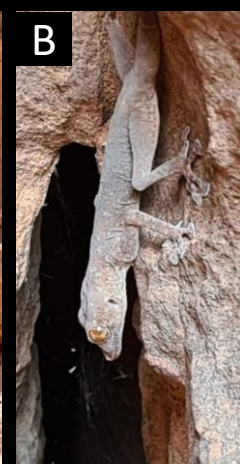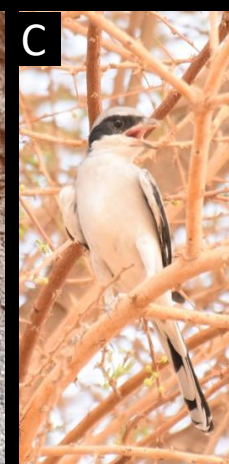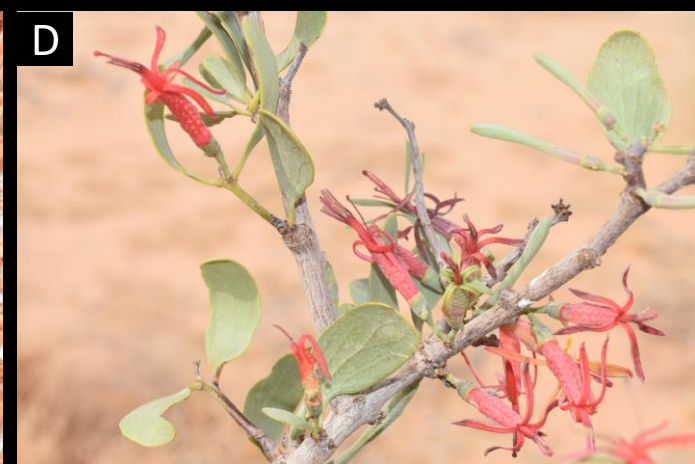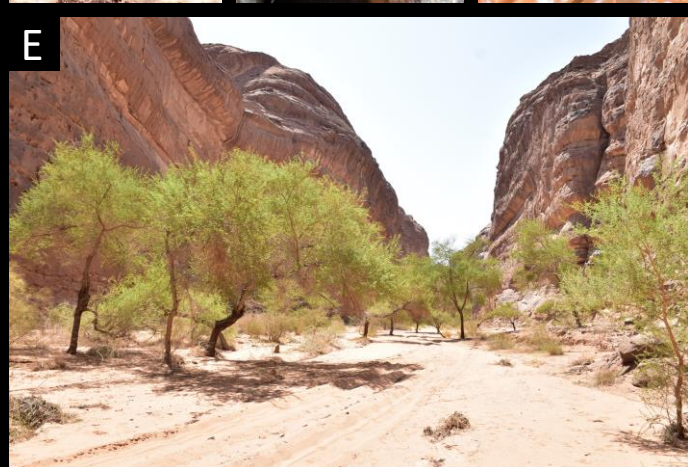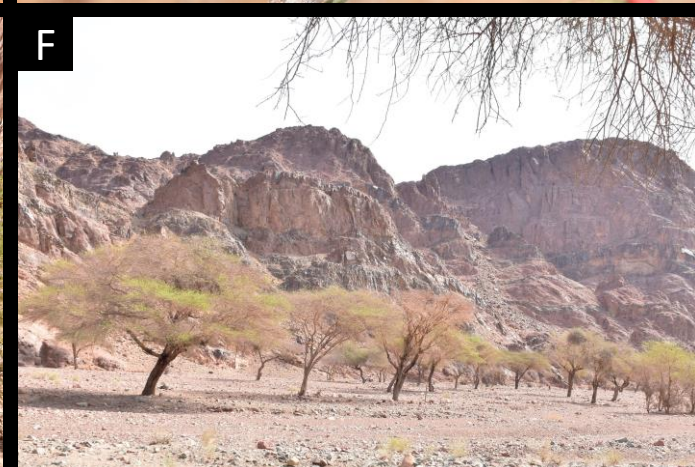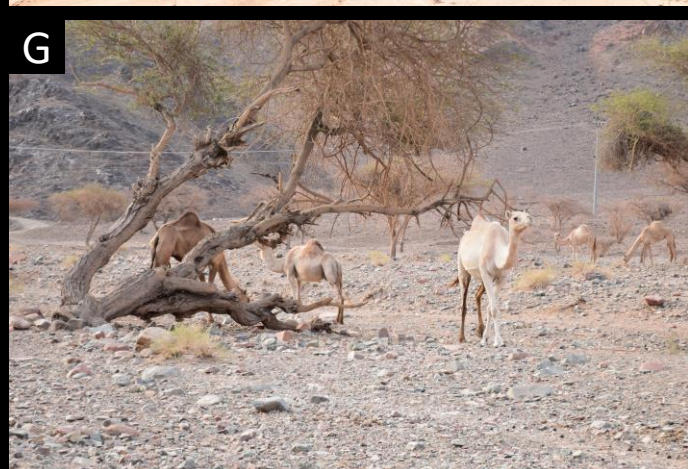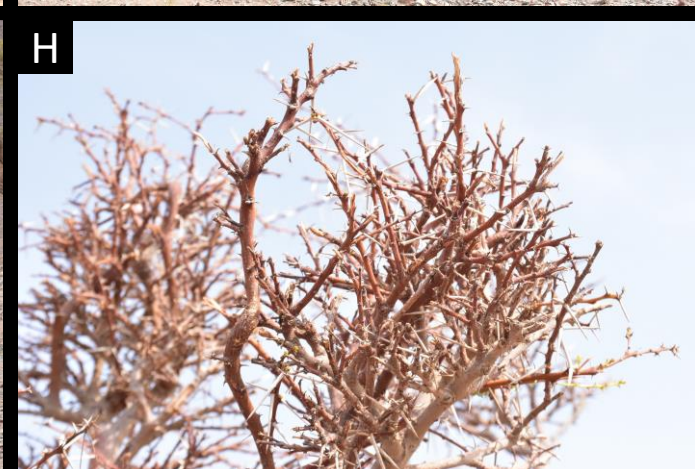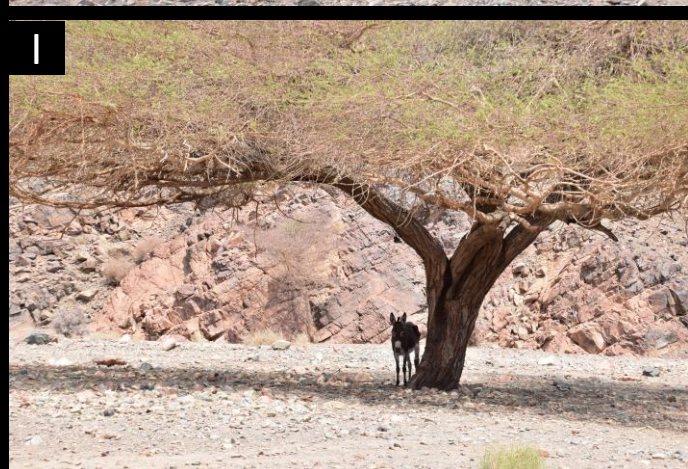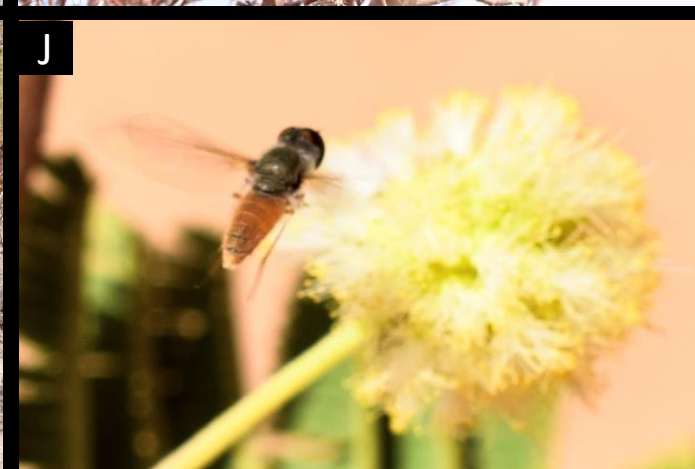

Supplement: Supplementary file 1 — Supplementary file1 (PDF 563 KB) [file 425_2024_4407_MOESM1_ESM.pdf]

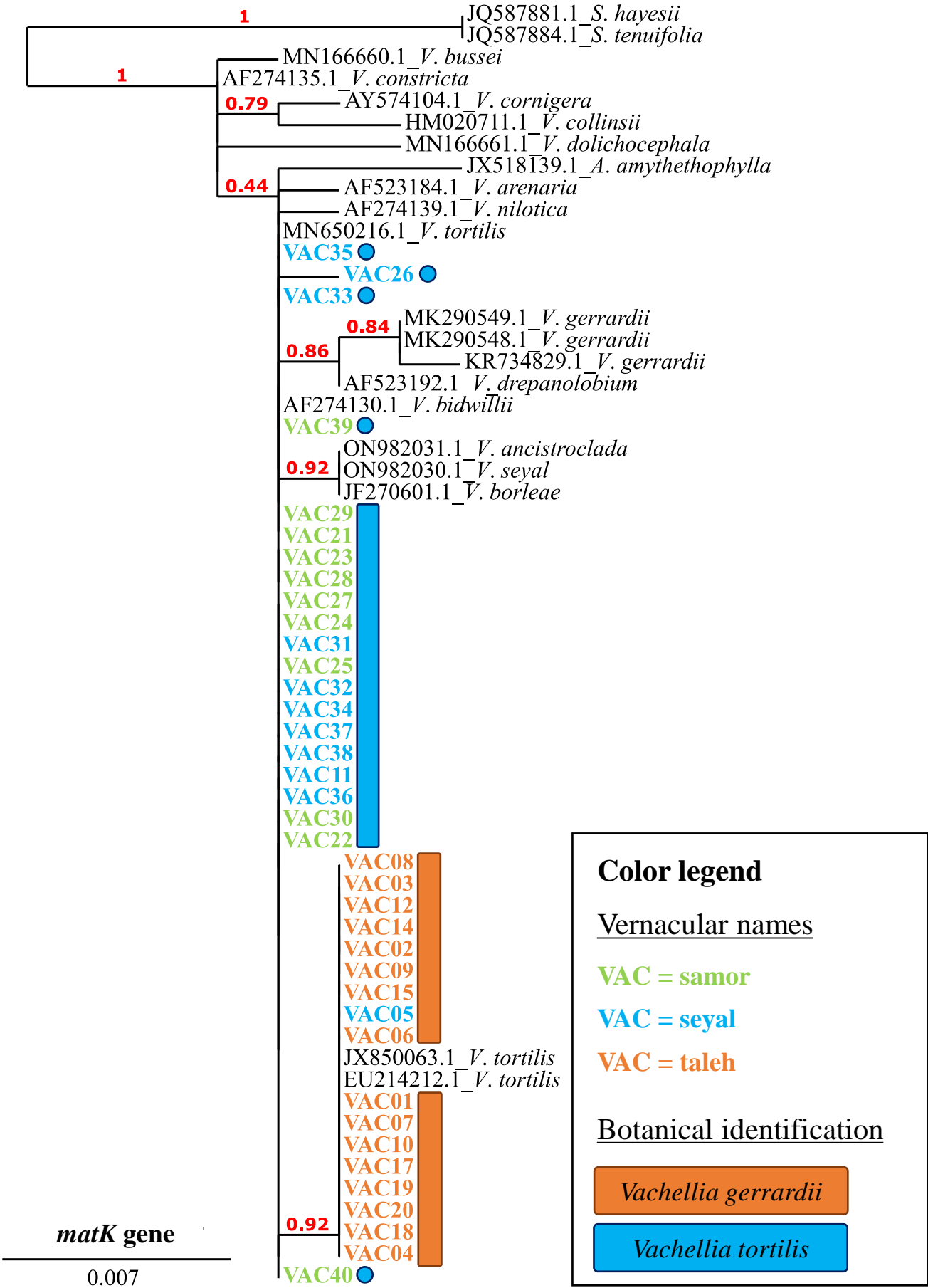

Supplement: Supplementary file 3 — Supplementary file3 (PDF 222 KB) [file 425_2024_4407_MOESM3_ESM.pdf]

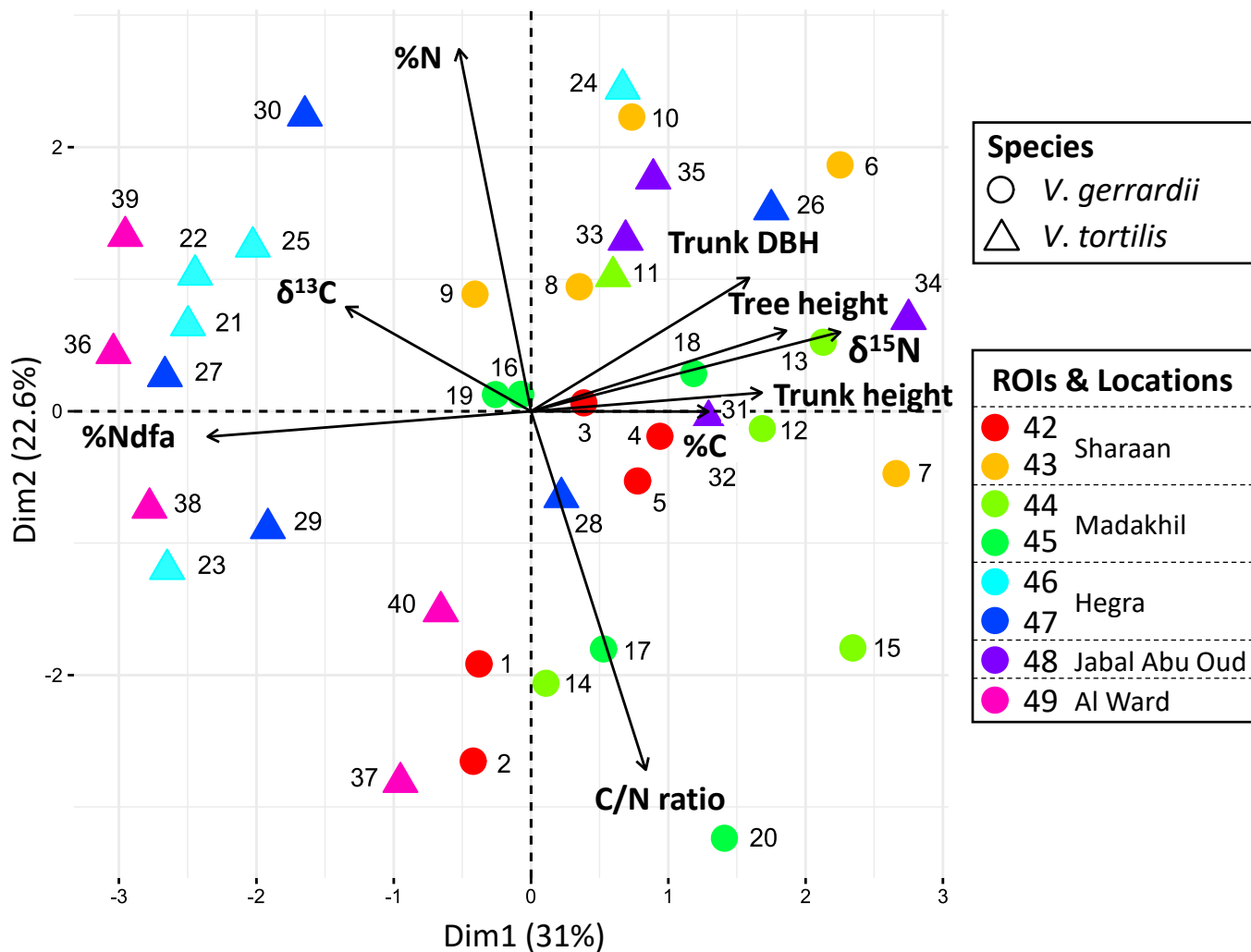

Supplement: Supplementary file 5 — Supplementary file5 (PDF 156 KB) [file 425_2024_4407_MOESM5_ESM.pdf]
