# Supplementary material for: Ecological aspects and relationships of the emblematic Vachellia spp. exposed to anthropic pressures and parasitism in natural hyper-arid ecosystems: ethnobotanical elements, morphology, and biological nitrogen fixation
Source: Planta. 2024 Apr 25;259(6):132. doi: 10.1007/s00425-024-04407-0 (PMC11045644; doi:10.1007/s00425-024-04407-0)
Supplement: Supplementary file 2 — Supplementary file2 (PDF 660 KB) [file 425_2024_4407_MOESM2_ESM.pdf]

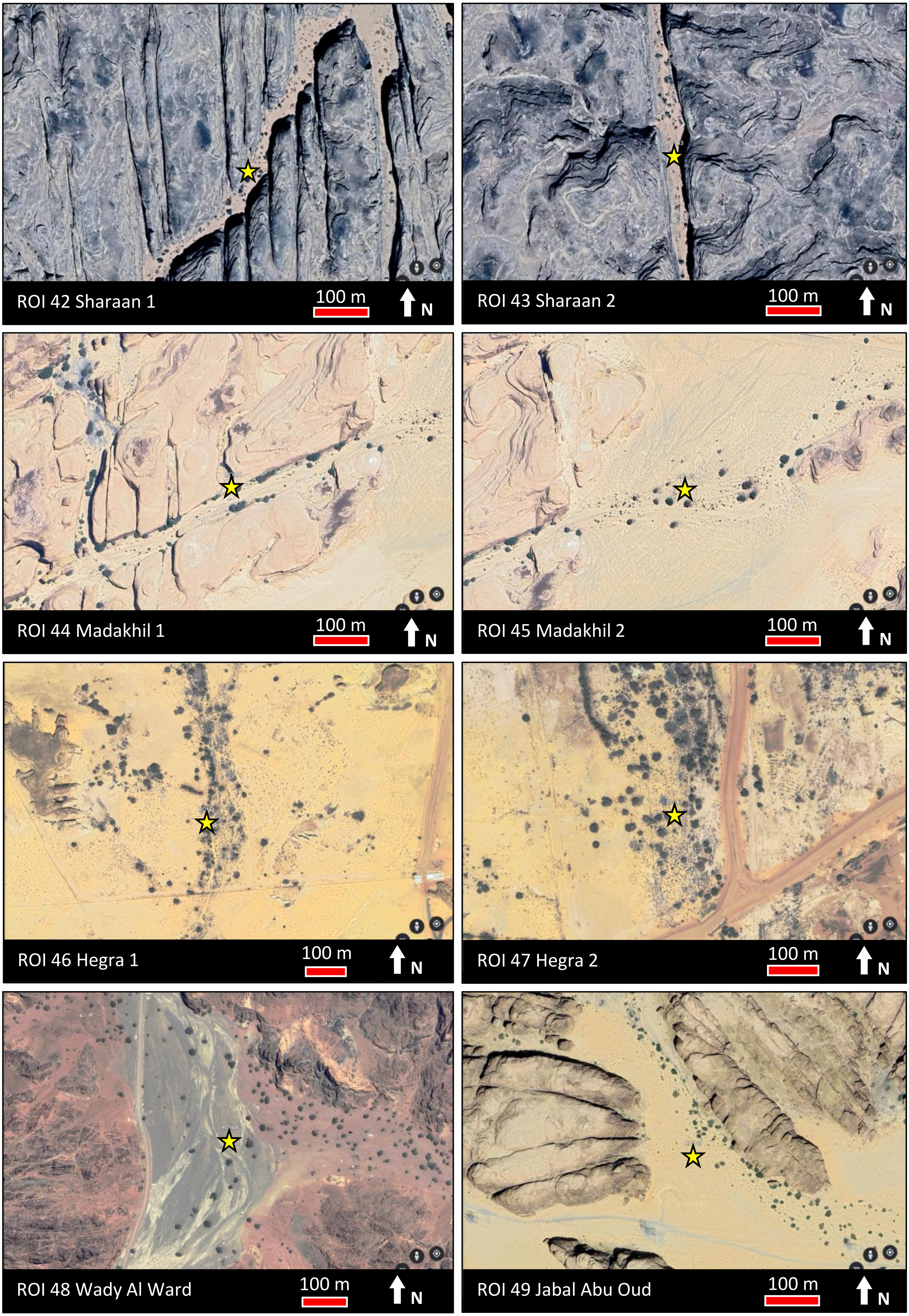
ROI 42 Sharaan 1
100 m
N
ROI 43 Sharaan 2
100 m
N
ROI 44 Madakhil 1
100 m
N
ROI 45 Madakhil 2
100 m
N
ROI 46 Hegra 1
100 m
N
ROI 47 Hegra 2
100 m
N
ROI 48 Wady Al Ward
100 m
N
ROI 49 Jabal Abu Oud
100 m
N
