# Supplementary material for: Ecological aspects and relationships of the emblematic Vachellia spp. exposed to anthropic pressures and parasitism in natural hyper-arid ecosystems: ethnobotanical elements, morphology, and biological nitrogen fixation
Source: Planta. 2024 Apr 25;259(6):132. doi: 10.1007/s00425-024-04407-0 (PMC11045644; doi:10.1007/s00425-024-04407-0)
Supplement: Supplementary file 4 — Supplementary file4 (PDF 269 KB) [file 425_2024_4407_MOESM4_ESM.pdf]

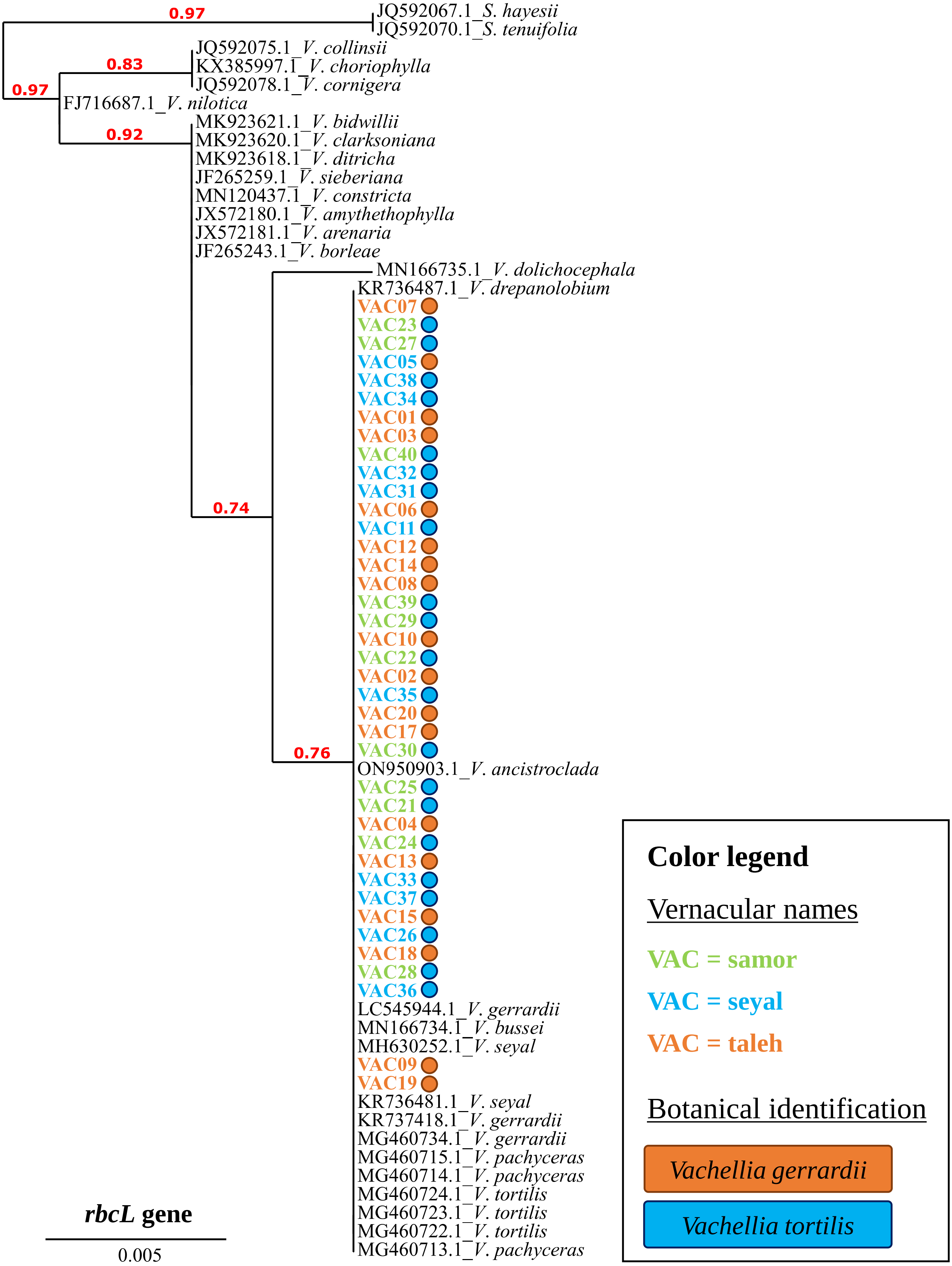
0.97
JQ592067.1_S. hayesii
JQ592070.1_S. tenuifolia
0.83
JQ592075.1_V. collinsii
KX385997.1_V. choriophylla
JQ592078.1_V. cornigera
0.97
FJ716687.1_V. nilotica
0.92
MK923621.1_V. bidwillii
MK923620.1_V. clarksoniana
MK923618.1_V. ditricha
JF265259.1_V. sieberiana
MN120437.1_V. constricta
JX572180.1_V. amythethophylla
JX572181.1_V. arenaria
JF265243.1_V. borleae
MN166735.1_V. dolichocephala
KR736487.1_V. drepanolobium
VAC07
VAC23
VAC27
VAC05
VAC38
VAC34
VAC01
VAC03
VAC40
VAC32
VAC31
0.74
VAC06
VAC11
VAC12
VAC14
VAC08
VAC39
VAC29
VAC10
VAC22
VAC02
VAC35
VAC20
VAC17
0.76
VAC30
ON950903.1_V. ancistroclada
VAC25
VAC21
VAC04
VAC24
VAC13
VAC33
VAC37
VAC15
VAC26
VAC18
VAC28
VAC36
LC545944.1_V. gerrardii
MN166734.1_V. bussei
MH630252.1_V. seyal
VAC09
VAC19
KR736481.1_V. seyal
KR737418.1_V. gerrardii
MG460734.1_V. gerrardii
MG460715.1_V. pachyceras
MG460714.1_V. pachyceras
MG460724.1_V. tortilis
MG460723.1_V. tortilis
MG460722.1_V. tortilis
MG460713.1_V. pachyceras
rbcL gene
0.005
Color legend
Vernacular names
VAC = samor
VAC = seyal
VAC = taleh
Botanical identification
Vachellia gerrardii
Vachellia tortilis
