# Supplementary material for: Ecological aspects and relationships of the emblematic Vachellia spp. exposed to anthropic pressures and parasitism in natural hyper-arid ecosystems: ethnobotanical elements, morphology, and biological nitrogen fixation
Source: Planta. 2024 Apr 25;259(6):132. doi: 10.1007/s00425-024-04407-0 (PMC11045644; doi:10.1007/s00425-024-04407-0)
Supplement: Supplementary file 6 — Supplementary file6 (PDF 91 KB) [file 425_2024_4407_MOESM6_ESM.pdf]

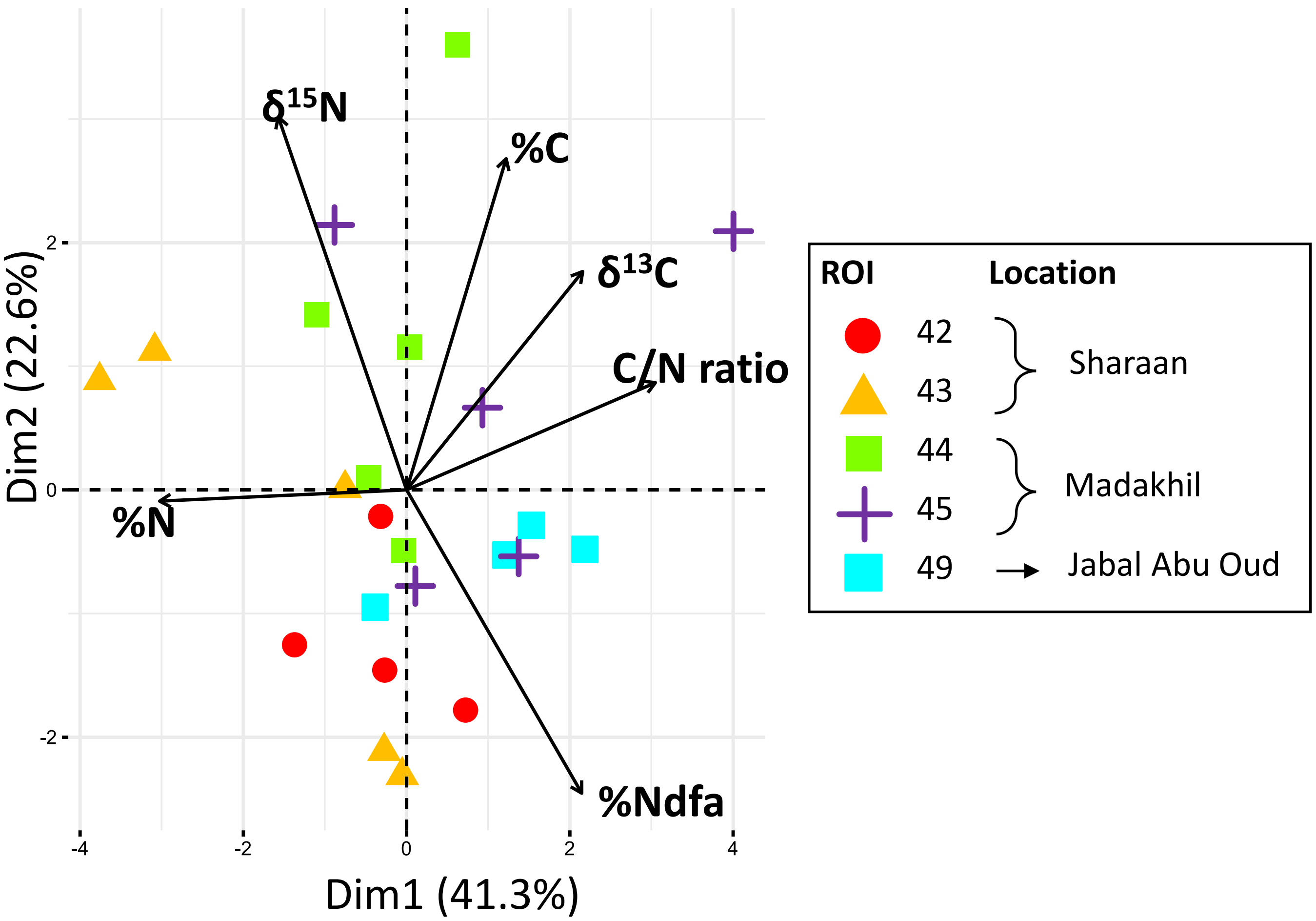

Fig. S6: Comparison of *Retama raetam* characteristics across ROIs, represented in a principal component analysis (PCA) plot.
