# Supplementary material for: Ecological aspects and relationships of the emblematic Vachellia spp. exposed to anthropic pressures and parasitism in natural hyper-arid ecosystems: ethnobotanical elements, morphology, and biological nitrogen fixation
Source: Planta. 2024 Apr 25;259(6):132. doi: 10.1007/s00425-024-04407-0 (PMC11045644; doi:10.1007/s00425-024-04407-0)
Supplement: Supplementary file 7 — Supplementary file7 (DOCX 13 KB) [file 425_2024_4407_MOESM7_ESM.docx]

**Fig. S1** Panel of pictures showing a fraction of the fauna and flora encountered in the AlUla region: *Upupa* *epops* individual (**a**); *Ptyodactylus* *hasselquistii* individual (**b**); *Lanius* sp. Individual (**c**); close-up of *Plicosepalus* *acaciae* flowers (**d**); healthy *Vachellia* population (**e**); non-healthy *Vachellia* population (**f**); *Camelus* *dromedarius* individuals most responsible of local overgrazing (**g**); heavily-grazed *Vachellia* branches (**h**); *Equus* *asinus* taking cover in the shade of *Vachellia* (**i**); a pollinator targeting a *Vachellia* flower (**j**)

**Fig. S2** Orthophotographic views of the eight regions of interest (ROI). Photos were obtained from Google Earth. Yellow stars represent the ROI coordinates (see Table 1)

**Fig. S3** Phylogenetic tree based on the maximum likelihood of *matK* genes from the *Vachellia* spp. sampled (in bold) among several reference species. Vernacular and botanical names after identification with the dichotomous key are indicated

**Fig. S4** Phylogenetic tree based on the maximum likelihood of *rcbL* genes from the *Vachellia* spp. sampled (in bold) among several reference species. Vernacular names and botanical names after identification with the dichotomous key are indicated

**Fig. S5** Characteristics of *Vachellia* trees (*V*. *gerrardii* or *V*. *tortilis*) and ROI location, represented in a principal component analysis (PCA) plot

**Fig. S6** Comparison of *Retama* *raetam* characteristics across ROIs, represented in a principal component analysis (PCA) plot
