# Supplementary material for: Ecological aspects and relationships of the emblematic Vachellia spp. exposed to anthropic pressures and parasitism in natural hyper-arid ecosystems: ethnobotanical elements, morphology, and biological nitrogen fixation
Source: Planta. 2024 Apr 25;259(6):132. doi: 10.1007/s00425-024-04407-0 (PMC11045644; doi:10.1007/s00425-024-04407-0)
Supplement: Supplementary file 8 — Supplementary file8 (DOCX 22 KB) [file 425_2024_4407_MOESM8_ESM.docx]

**Table S1** *Vachellia* tree characteristics, including species identification, locations, health status (Health 1 = detailed status; Health 2 = condensed version), and plant morphologic traits. The Health 2 class includes healthy trees and healthy-parasitized trees. The non-healthy class included parasitized trees, grazed trees and parasitized/grazed trees

| **Tree number** | **Latin name** | **Common name** | **ROI number** | **Land use** | **Health 1** | **Health 2** | **Tree height (m)** | **Trunk height (m)** | **Trunk**  **DBH (cm)** |
| --- | --- | --- | --- | --- | --- | --- | --- | --- | --- |
| VAC 01 | *V*. *gerrardii* | Taleh | ROI 42 | Natural reserve | Healthy | Healthy | 6.4 | 3.4 | 92 |
| VAC 02 | *V*. *gerrardii* | Taleh | ROI 42 | Natural reserve | Healthy | Healthy | 7 | 3.96 | 88 |
| VAC 03 | *V*. *gerrardii* | Taleh | ROI 42 | Natural reserve | Parasitized | Non-Healthy | 10 | 2.8 | 123 |
| VAC 04 | *V*. *gerrardii* | Taleh | ROI 42 | Natural reserve | Healthy - Parasitized | Healthy | 8 | 2.3 | 184 |
| VAC 05 | *V*. *gerrardii* | Seyal | ROI 42 | Natural reserve | Parasitized | Non-Healthy | 8 | 2.6 | 114 |
| VAC 06 | *V*. *gerrardii* | Taleh | ROI 43 | Natural reserve | Healthy | Healthy | 10.3 | 3.5 | 115 |
| VAC 07 | *V*. *gerrardii* | Taleh | ROI 43 | Natural reserve | Healthy | Healthy | 12 | 3 | 120 |
| VAC 08 | *V*. *gerrardii* | Taleh | ROI 43 | Natural reserve | Parasitized | Non-Healthy | 14 | 3 | 100 |
| VAC 09 | *V*. *gerrardii* | Taleh | ROI 43 | Natural reserve | Parasitized | Non-Healthy | 8 | 3.3 | 125 |
| VAC 10 | *V*. *gerrardii* | Taleh | ROI 43 | Natural reserve | Healthy | Healthy | 6.5 | 3 | 90 |
| VAC 11 | *V. tortilis* | Seyal | ROI 44 | Natural reserve | Healthy | Healthy | 10 | 3 | 145 |
| VAC 12 | *V*. *gerrardii* | Taleh | ROI 44 | Natural reserve | Healthy | Healthy | 8 | 3 | 91 |
| VAC 13 | *V*. *gerrardii* | Taleh | ROI 44 | Natural reserve | Healthy | Healthy | 10 | 5 | 200 |
| VAC 14 | *V*. *gerrardii* | Taleh | ROI 44 | Natural reserve | Healthy - Parasitized | Healthy | 5 | 2 | 104 |
| VAC 15 | *V*. *gerrardii* | Taleh | ROI 44 | Natural reserve | Healthy | Healthy | 8 | 4 | 100 |
| VAC 16 | *V*. *gerrardii* | Taleh | ROI 45 | Natural reserve | Healthy | Healthy | 8 | 2.3 | 150 |
| VAC 17 | *V*. *gerrardii* | Taleh | ROI 45 | Natural reserve | Healthy | Healthy | 7.5 | 2.5 | 120 |
| VAC 18 | *V*. *gerrardii* | Taleh | ROI 45 | Natural reserve | Healthy - Parasitized | Healthy | 9 | 2.6 | 170 |
| VAC 19 | *V*. *gerrardii* | Taleh | ROI 45 | Natural reserve | Healthy - Parasitized | Healthy | 5 | 1.3 | 155 |
| VAC 20 | *V*. *gerrardii* | Taleh | ROI 45 | Natural reserve | Healthy - Parasitized | Healthy | 5 | 2.8 | 85 |
| VAC 21 | *V. tortilis* | Samor | ROI 46 | Archeologic site | Parasitized | Non-Healthy | 5 | 2 | 55 |
| VAC 22 | *V. tortilis* | Samor | ROI 46 | Archeologic site | Parasitized | Non-Healthy | 5 | 2 | 60 |
| VAC 23 | *V. tortilis* | Samor | ROI 46 | Archeologic site | Parasitized | Non-Healthy | 5.5 | 2 | 70 |
| VAC 24 | *V. tortilis* | Samor | ROI 46 | Archeologic site | Parasitized | Non-Healthy | 6.6 | 2.5 | 286 |
| VAC 25 | *V. tortilis* | Samor | ROI 46 | Archeologic site | Parasitized | Non-Healthy | 6 | 2.4 | 160 |
| VAC 26 | *V. tortilis* | Seyal | ROI 47 | Archeologic site | Healthy | Healthy | 11 | 4 | 270 |
| VAC 27 | *V. tortilis* | Samor | ROI 47 | Archeologic site | Healthy | Healthy | 6.5 | 2 | 60 |
| VAC 28 | *V. tortilis* | Samor | ROI 47 | Archeologic site | Healthy | Healthy | 7 | 2 | 150 |
| VAC 29 | *V. tortilis* | Samor | ROI 47 | Archeologic site | Healthy | Healthy | 7 | 2 | 80 |
| VAC 30 | *V. tortilis* | Samor | ROI 47 | Archeologic site | Healthy | Healthy | 6 | 2.5 | 90 |
| VAC 31 | *V. tortilis* | Seyal | ROI 48 | Public domain | Parasitized - Grazed | Non-Healthy | 7.5 | 2.5 | 210 |
| VAC 32 | *V. tortilis* | Seyal | ROI 48 | Public domain | Grazed | Non-Healthy | 7 | 2 | 265 |
| VAC 33 | *V. tortilis* | Seyal | ROI 48 | Public domain | Grazed | Non-Healthy | 6 | 2 | 175 |
| VAC 34 | *V. tortilis* | Seyal | ROI 48 | Public domain | Parasitized - Grazed | Non-Healthy | 8 | 3 | 235 |
| VAC 35 | *V. tortilis* | Seyal | ROI 48 | Public domain | Grazed | Non-Healthy | 8.6 | 4 | 235 |
| VAC 36 | *V. tortilis* | Seyal | ROI 49 | Public domain | Parasitized - Grazed | Non-Healthy | 5 | 2.5 | 62 |
| VAC 37 | *V. tortilis* | Seyal | ROI 49 | Public domain | Parasitized - Grazed | Non-Healthy | 6 | 3 | 115 |
| VAC 38 | *V. tortilis* | Seyal | ROI 49 | Public domain | Parasitized - Grazed | Non-Healthy | 4 | 1 | 70 |
| VAC 39 | *V. tortilis* | Samor | ROI 49 | Public domain | Parasitized - Grazed | Non-Healthy | 4 | 1.5 | 129 |
| VAC 40 | *V. tortilis* | Samor | ROI 49 | Public domain | Parasitized - Grazed | Non-Healthy | 7.2 | 1.5 | 145 |
