# Supplementary material for: Ecological aspects and relationships of the emblematic Vachellia spp. exposed to anthropic pressures and parasitism in natural hyper-arid ecosystems: ethnobotanical elements, morphology, and biological nitrogen fixation
Source: Planta. 2024 Apr 25;259(6):132. doi: 10.1007/s00425-024-04407-0 (PMC11045644; doi:10.1007/s00425-024-04407-0)
Supplement: Supplementary file 9 — Supplementary file9 (DOCX 14 KB) [file 425_2024_4407_MOESM9_ESM.docx]

**Table S2** Health status of *Vachellia* individuals in relation to: the land use (**a**), their morphological traits (**b**) and their species (**c**). Statistical tests used: chi² (**a** and **c**) and Wilcoxon-Mann-Whitney tests (**b**). The values given are either the number of trees (**a** and **c**), or the mean ± standard error (**b**). Means with the same letter are considered similar (at *P* < 0.05)

| **Land use (a)** | **Natural**  **reserve** | **Archaeological site** | **Public domain** | Chi² test |
| --- | --- | --- | --- | --- |
| Healthy (*n*=21) | 16 | 5 | 0 | X² = 17.1*** |
| Non-healthy (*n*=19) | 4 | 5 | 10 |  |
|  |  |  |  |  |
| **Tree morphology (b)** | **Tree height (m)** | **Trunk height (m)** | **Trunk DBH (cm)** |  |
| Healthy (*n*=21) | 7.8 ± 2.0 a | 2.9 ± 0.9 a | 127 ± 50 a |  |
| Non-healthy (*n*=19) | 6.9 ± 2.3 a | 2.4 ± 0.7 a | 144 ± 72 a |  |
|  |  |  |  |  |
| ***Vachellia* species (c)** | ***V. gerrardii*** | ***V. tortilis*** | Chi² test |  |
| Healthy (*n*=21) | 15 | 6 | X²= 8.2** |  |
| Non-healthy (*n*=19) | 4 | 15 |  |  |
