# Supplementary material for: Ecological aspects and relationships of the emblematic Vachellia spp. exposed to anthropic pressures and parasitism in natural hyper-arid ecosystems: ethnobotanical elements, morphology, and biological nitrogen fixation
Source: Planta. 2024 Apr 25;259(6):132. doi: 10.1007/s00425-024-04407-0 (PMC11045644; doi:10.1007/s00425-024-04407-0)
Supplement: Supplementary file 10 — Supplementary file10 (DOCX 15 KB) [file 425_2024_4407_MOESM10_ESM.docx]

**Table S3** Accession numbers of the leguminous reference sequences retrieved on NCBI for *matK* and *rbcL* genes. Multiple sequences were retrieved for *V*. *gerrardii*, *V*. *pachyceras*, *V*. *tortilis* and *V*. *seyal*

|  | **Accession number** |  |
| --- | --- | --- |
| **Plant species** | ***matK* gene** | ***rbcL* gene** |
| *Senegallia hayesii* | JQ587881.1 | JQ592067.1 |
| *Senegallia tenuifolia* | JQ587884.1 | JQ592070.1 |
| *Vachellia amythetophylla* | JX518139.1 | JX572180.1 |
| *Vachellia ancistroclada* | ON982031.1 | ON950903.1 |
| *Vachellia arenaria* | AF523184.1 | JX572181.1 |
| *Vachellia bidwillii* | AF274130.1 | MK923621.1 |
| *Vachellia borleae* | JF270601.1 | JF265243.1 |
| *Vachellia bussei* | MN166660.1 | MN166734.1 |
| *Vachellia choriophylla* | - | KX385997.1 |
| *Vachellia clarksoniana* | - | MK923620.1 |
| *Vachellia collinsii* | HM020711.1 | JQ592075.1 |
| *Vachellia constricta* | AF274135.1 | MN120437.1 |
| *Vachellia cornigera* | AY574104.1 | JQ592078.1 |
| *Vachellia ditricha* | - | MK923618.1 |
| *Vachellia dolichocephala* | MN166661.1 | MN166735.1 |
| *Vachellia drepanolobium* | AF523192.1 | KR736487.1 |
| *Vachellia gerrardii* 1 | MK290549.1 | LC545944.1 |
| *Vachellia gerrardii* 2 | MK290548.1 | KR737418.1 |
| *Vachellia gerrardii* 3 | KR734829.1 | MG460734.1 |
| *Vachellia nilotica* | AF274139.1 | FJ716687.1 |
| *Vachellia pachyceras* 1 | - | MG460715.1 |
| *Vachellia pachyceras* 2 | - | MG460714.1 |
| *Vachellia pachyceras* 3 | - | MG460713.1 |
| *Vachellia seyal* 1 | ON982030.1 | MH630252.1 |
| *Vachellia seyal* 2 | - | KR736481.1 |
| *Vachellia sieberiana* | - | JF265259.1 |
| *Vachellia tortilis* 1 | MN650216.1 | MG460722.1 |
| *Vachellia tortilis* 2 | JX850063.1 | MG460723.1 |
| *Vachellia tortilis* 3 | EU214212.1 | MG460724.1 |
