# Supplementary material for: Ecological aspects and relationships of the emblematic Vachellia spp. exposed to anthropic pressures and parasitism in natural hyper-arid ecosystems: ethnobotanical elements, morphology, and biological nitrogen fixation
Source: Planta. 2024 Apr 25;259(6):132. doi: 10.1007/s00425-024-04407-0 (PMC11045644; doi:10.1007/s00425-024-04407-0)
Supplement: Supplementary file 11 — Supplementary file11 (DOCX 17 KB) [file 425_2024_4407_MOESM11_ESM.docx]

**Table S4** Top three nucleotide BLAST results for *matK* genes from *Vachellia* spp. trees. Results are given with the species name, percentage of identity between the sample and the reference sequences with their corresponding accession numbers

| **Region of interest** | **Tree number** | **First result** | **Second result** | **Third result** |
| --- | --- | --- | --- | --- |
| ROI 42 | VAC 01 | *Acacia* *iraqensis* (100%) LC552610 | *Vachellia* *gerrardii* var. *gerrardii* (100%) LC552597 | *Vachellia* *gerrardii* var. *najdensis* (100%) LC552592 |
| ROI 42 | VAC 02 | *Acacia* *iraqensis* (99.73%) LC552610 | *Vachellia* *gerrardii* var. *gerrardii* (99.73%) LC552597 | *Vachellia* *gerrardii* var. *najdensis* (99.73%) LC552592 |
| ROI 42 | VAC 03 | *Acacia* *iraqensis* (99.32%) LC552610 | *Vachellia* *gerrardii* var. *gerrardii* (99.32%) LC552597 | *Vachellia* *gerrardii* var. najdensis (99.32%) LC552592 |
| ROI 42 | VAC 04 | *Acacia* *iraqensis* (100%) LC552610 | *Vachellia gerrardii* var. *gerrardii* (100%) LC552597 | *Vachellia* *gerrardii* var. *najdensis* (100%) LC552592 |
| ROI 42 | VAC 05 | *Acacia* *iraqensis* (99.87%) LC552610 | *Vachellia* *gerrardii* var. *gerrardii* (99.87%) LC552597 | *Vachellia* *gerrardii* var. *najdensis* (99.87%) LC552592 |
| ROI 43 | VAC 06 | *Acacia* *iraqensis* (99.73%) LC552610 | *Vachellia gerrardii* var. *gerrardii* (99.73%) LC552597 | *Vachellia gerrardii* var. *najdensis* (99.73%) LC552592 |
| ROI 43 | VAC 07 | *Acacia* *iraqensis* (100%) LC552610 | *Vachellia gerrardii* var. *gerrardii* (100%) LC552597 | *Vachellia gerrardii* var. *najdensis* (100%) LC552592 |
| ROI 43 | VAC 08 | *Acacia* *iraqensis* (99.21%) LC552610 | *Vachellia gerrardii* var. *gerrardii* (99.21%) LC552597 | *Vachellia gerrardii* var. *najdensis* (99.21%) LC552592 |
| ROI 43 | VAC 09 | *Acacia* *iraqensis* (99.74%) LC552610 | *Vachellia gerrardii* var. *gerrardii* (99.74%) LC552597 | *Vachellia gerrardii* var. *najdensis* (99.74%) LC552592 |
| ROI 43 | VAC 10 | *Acacia iraqensis* (100%) LC552610 | *Vachellia gerrardii* var. *gerrardii* (100%) LC552597 | *Vachellia gerrardii* var. *najdensis* (100%) LC552592 |
| ROI 44 | VAC 11 | *Vachellia tortilis* subsp. *raddiana* (99.86%) LC552605 | *Vachellia tortilis* subsp. *tortilis* (99.86%) LC552589 | *Vachellia tortilis* isolate UHURU1133-14 (99.86%) MK290437 |
| ROI 44 | VAC 12 | *Acacia iraqensis* (99.31%) LC552610 | *Vachellia gerrardii* var. *gerrardii* (99.31%) LC552597 | *Vachellia gerrardii* var. *najdensis* (99.31%) LC552592 |
| ROI 44 | VAC 13 | NONE | NONE | NONE |
| ROI 44 | VAC 14 | *Acacia iraqensis* (99.47%) LC552610 | *Vachellia gerrardii* var. *gerrardii* (99.47%) LC552597 | *Vachellia gerrardii* var. najdensis (99.47%) LC552592 |
| ROI 44 | VAC 15 | *Acacia iraqensis* (99.60%) LC552610 | *Vachellia gerrardii* var. *gerrardii* (99.60%) LC552597 | Vachellia gerrardii var. najdensis (99.60%) LC552592 |
| ROI 45 | VAC 16 | NONE | NONE | NONE |
| ROI 45 | VAC 17 | *Acacia iraqensis* (100%) LC552610 | *Vachellia gerrardii* var. *gerrardii* (100%) LC552597 | *Vachellia gerrardii* var. *najdensis* (100%) LC552592 |
| ROI 45 | VAC 18 | *Acacia iraqensis* (100%) LC552610 | *Vachellia gerrardii* var. *gerrardii* (100%) LC552597 | *Vachellia gerrardii* var. *najdensis* (100%) LC552592 |
| ROI 45 | VAC 19 | *Acacia iraqensis* (100%) LC552610 | *Vachellia gerrardii* var. *gerrardii* (100%) LC552597 | *Vachellia gerrardii* var. *najdensis* (100%) LC552592 |
| ROI 45 | VAC 20 | *Acacia iraqensis* (100%) LC552610 | *Vachellia gerrardii* var. *gerrardii* (100%) LC552597 | *Vachellia gerrardii* var. *najdensis* (100%) LC552592 |
| ROI 46 | VAC 21 | *Vachellia tortilis* subsp. *raddiana* (99.60%) LC552605 | *Vachellia tortilis* subsp. *tortilis* (99.60%) LC552589 | *Vachellia tortilis* isolate UHURU1133-14 (99.60%) MK290437 |
| ROI 46 | VAC 22 | *Vachellia tortilis* subsp. *raddiana* (99.74%) LC552605 | *Vachellia tortilis* subsp. *tortilis* (99.74%) LC552589 | *Vachellia tortilis* isolate UHURU1133-14 (99.74%) MK290437 |
| ROI 46 | VAC 23 | *Vachellia tortilis* subsp. *raddiana* (99.87%) LC552605 | *Vachellia tortilis* subsp. *tortilis* (99.87%) LC552589 | *Vachellia tortilis* isolate UHURU1133-14 (99.87%) MK290437 |
| ROI 46 | VAC 24 | *Vachellia tortilis* subsp. *raddiana* (100%) LC552605 | *Vachellia tortilis* subsp. *tortilis* (100%) LC552589 | *Vachellia tortilis* isolate UHURU1133-14 (100%) MK290437 |
| ROI 46 | VAC 25 | *Vachellia tortilis* subsp. *raddiana* (99.74%) LC552605 | *Vachellia tortilis* subsp. *tortilis* (99.74%) LC552589 | *Vachellia tortilis* isolate UHURU1133-14 (99.74%) MK290437 |
| ROI 47 | VAC 26 | *Vachellia tortilis* subsp. *raddiana* (99.74%) LC552605 | *Vachellia tortilis* subsp. *tortilis* (99.74%) LC552589 | *Vachellia tortilis* isolate UHURU1133-14 (99.74%) MK290437 |
| ROI 47 | VAC 27 | *Vachellia tortilis* subsp. *raddiana* (100%) LC552605 | *Vachellia tortilis* subsp. *tortilis* (100%) LC552589 | *Vachellia tortilis* isolate UHURU1133-14 (100%) MK290437 |
| ROI 47 | VAC 28 | *Vachellia tortilis* subsp. *raddiana* (100%) LC552605 | *Vachellia tortilis* subsp. *tortilis* (100%) LC552589 | *Vachellia tortilis* isolate UHURU1133-14 (100%) MK290437 |
| ROI 47 | VAC 29 | *Vachellia tortilis* subsp. *raddiana* (100%) LC552605 | *Vachellia tortilis* subsp. *tortilis* (100%) LC552589 | *Vachellia tortilis* isolate UHURU1133-14 (100%) MK290437 |
| ROI 47 | VAC 30 | *Vachellia tortilis* subsp. *raddiana* (100%) LC552605 | *Vachellia tortilis* subsp. *tortilis* (100%) LC552589 | *Vachellia tortilis* isolate UHURU1133-14 (100%) MK290437 |
| ROI 48 | VAC 31 | *Vachellia tortilis* subsp. *raddiana* (99.87%) LC552605 | *Vachellia tortilis* subsp. *tortilis* (99.87%) LC552589 | *Vachellia tortilis* isolate UHURU1133-14 (99.87%) MK290437 |
| ROI 48 | VAC 32 | *Vachellia tortilis* subsp. *raddiana* (99.86%) LC552605 | *Vachellia tortilis* subsp. *tortilis* (99.86%) LC552589 | *Vachellia tortilis* isolate UHURU1133-14 (99.86%) MK290437 |
| ROI 48 | VAC 33 | *Vachellia tortilis* subsp. *raddiana* (100%) LC552605 | *Vachellia tortilis* subsp. *tortilis* (100%) LC552589 | *Vachellia tortilis* isolate UHURU1133-14 (100%) MK290437 |
| ROI 48 | VAC 34 | *Vachellia tortilis* subsp. *raddiana* (99.86%) LC552605 | *Vachellia tortilis* subsp. *tortilis* (99.86%) LC552589 | *Vachellia tortilis* isolate UHURU1133-14 (99.86%) MK290437 |
| ROI 48 | VAC 35 | *Vachellia tortilis* subsp. *raddiana* (100%) LC552605 | *Vachellia tortilis* subsp. *tortilis* (100%) LC552589 | *Vachellia tortilis* isolate UHURU1133-14 (100%) MK290437 |
| ROI 49 | VAC 36 | *Vachellia tortilis* subsp. *raddiana* (100%) LC552605 | *Vachellia tortilis* subsp. *tortilis* (100%) LC552589 | *Vachellia tortilis* isolate UHURU1133-14 (100%) MK290437 |
| ROI 49 | VAC 37 | *Vachellia tortilis* subsp. *raddiana* (99.86%) LC552605 | *Vachellia tortilis* subsp. *tortilis* (99.86%) LC552589 | *Vachellia tortilis* isolate UHURU1133-14 (99.86%) MK290437 |
| ROI 49 | VAC 38 | *Vachellia tortilis* subsp. *raddiana* (100%) LC552605 | *Vachellia tortilis* subsp. *tortilis* (100%) LC552589 | *Vachellia tortilis* isolate UHURU1133-14 (100%) MK290437 |
| ROI 49 | VAC 39 | *Vachellia tortilis* subsp. *raddiana* (100%) LC552605 | *Vachellia tortilis* subsp. *tortilis* (100%) LC552589 | *Vachellia tortilis* isolate UHURU1133-14 (100%) MK290437 |
| ROI 49 | VAC 40 | *Vachellia tortilis* subsp. *raddiana* (100%) LC552605 | *Vachellia tortilis* subsp. *tortilis* (100%) LC552589 | *Vachellia tortilis* isolate UHURU1133-14 (100%) MK290437 |
