# Supplementary material for: Ecological aspects and relationships of the emblematic Vachellia spp. exposed to anthropic pressures and parasitism in natural hyper-arid ecosystems: ethnobotanical elements, morphology, and biological nitrogen fixation
Source: Planta. 2024 Apr 25;259(6):132. doi: 10.1007/s00425-024-04407-0 (PMC11045644; doi:10.1007/s00425-024-04407-0)
Supplement: Supplementary file 12 — Supplementary file12 (DOCX 17 KB) [file 425_2024_4407_MOESM12_ESM.docx]

**Table S5** Top three nucleotide BLAST results for *rbcL* genes from *Vachellia* spp. trees. Results are given with the species name, percentage of identity between the sample and the reference sequences with their corresponding accession numbers

| **Region of interest** | **Tree number** | **First result** | **Second result** | **Third result** |
| --- | --- | --- | --- | --- |
| ROI 42 | VAC 01 | *Senegalia* *senegal* (99.86%) NC045513 | *Vachelia* *tortilis* subsp. *raddiana* (99.86%) KY100266 | *Vachelia* *tortilis* subsp. *raddiana* (99.86%) KY100265 |
| ROI 42 | VAC 02 | *Senegalia* *senegal* (99.72%) NC045513 | *Vachelia* *tortilis* subsp. *raddiana* (99.72%) KY100266 | *Vachelia* *tortilis* subsp. *raddiana* (99.72%) KY100265 |
| ROI 42 | VAC 03 | *Senegalia* *senegal* (99.86%) NC045513 | *Vachelia* *tortilis* subsp. *raddiana* (99.86%) KY100266 | *Vachelia* *tortilis* subsp. *raddiana* (99.86%) KY100265 |
| ROI 42 | VAC 04 | *Senegalia* *senegal* (99.72%) NC045513 | *Vachelia* *tortilis* subsp. *raddiana* (99.72%) KY100266 | *Vachelia* *tortilis* subsp. *raddiana* (99.72%) KY100265 |
| ROI 42 | VAC 05 | *Senegalia* *senegal* (99.86%) NC045513 | *Vachelia* *tortilis* subsp. *raddiana* (99.86%) KY100266 | *Vachelia* *tortilis* subsp. *raddiana* (99.86%) KY100265 |
| ROI 43 | VAC 06 | *Senegalia* *senegal* (99.86%) NC045513 | *Vachelia* *tortilis* subsp. *raddiana* (99.86%) KY100266 | *Vachelia* *tortilis* subsp. *raddiana* (99.86%) KY100265 |
| ROI 43 | VAC 07 | *Senegalia* *senegal* (99.72%) NC045513 | *Vachelia* *tortilis* subsp. *raddiana* (99.72%) KY100266 | *Vachelia* *tortilis* subsp. *raddiana* (99.72%) KY100265 |
| ROI 43 | VAC 08 | *Senegalia* *senegal* (99.72%) NC045513 | *Vachelia* *tortilis* subsp. *raddiana* (99.72%) KY100266 | *Vachelia* *tortilis* subsp. *raddiana* (99.72%) KY100265 |
| ROI 43 | VAC 09 | *Senegalia* *senegal* (94.50%) NC045513 | *Vachelia* *tortilis* subsp. *raddiana* (94.50%) KY100266 | *Vachelia* *tortilis* subsp. *raddiana* (94.50%) KY100265 |
| ROI 43 | VAC 10 | *Senegalia* *senegal* (94.65%) NC045513 | *Vachelia* *tortilis* subsp. *raddiana* (94.65%) KY100266 | *Vachelia* *tortilis* subsp. *raddiana* (94.65%) KY100265 |
| ROI 44 | VAC 11 | *Senegalia* *senegal* (99.58%) NC045513 | *Vachelia* *tortilis* subsp. *raddiana* (99.58%) KY100266 | *Vachelia* *tortilis* subsp. *raddiana* (99.58%) KY100265 |
| ROI 44 | VAC 12 | *Senegalia* *senegal* (99.86%) NC045513 | *Vachelia* *tortilis* subsp. *raddiana* (99.86%) KY100266 | *Vachelia* *tortilis* subsp. *raddiana* (99.86%) KY100265 |
| ROI 44 | VAC 13 | *Senegalia* *senegal* (99.85%) NC045513 | *Vachelia* *tortilis* subsp. *raddiana* (99.85%) KY100266 | *Vachelia* *tortilis* subsp. *raddiana* (99.85%) KY100265 |
| ROI 44 | VAC 14 | *Senegalia* *senegal* (99.86%) NC045513 | *Vachelia* *tortilis* subsp. *raddiana* (99.86%) KY100266 | *Vachelia* *tortilis* subsp. *raddiana* (99.86%) KY100265 |
| ROI 44 | VAC 15 | *Senegalia* *senegal* (99.58%) NC045513 | *Vachelia* *tortilis* subsp. *raddiana* (99.58%) KY100266 | *Vachelia* *tortilis* subsp. *raddiana* (99.58%) KY100265 |
| ROI 45 | VAC 16 | NONE | NONE | NONE |
| ROI 45 | VAC 17 | *Senegalia* *senegal* (99.86%) NC045513 | *Vachelia* *tortilis* subsp. *raddiana* (99.86%) KY100266 | *Vachelia* *tortilis* subsp. *raddiana* (99.86%) KY100265 |
| ROI 45 | VAC 18 | *Senegalia* *senegal* (99.30%) NC045513 | *Vachelia* *tortilis* subsp. *raddiana* (99.30%) KY100266 | *Vachelia* *tortilis* subsp. *raddiana* (99.30%) KY100265 |
| ROI 45 | VAC 19 | *Senegalia* *senegal* (99.56%) NC045513 | *Vachelia* *tortilis* subsp. *raddiana* (99.56%) KY100266 | *Vachelia* *tortilis* subsp. *raddiana* (99.56%) KY100265 |
| ROI 45 | VAC 20 | *Senegalia* *senegal* (99.86%) NC045513 | *Vachelia* *tortilis* subsp. *raddiana* (99.86%) KY100266 | *Vachelia* *tortilis* subsp. *raddiana* (99.86%) KY100265 |
| ROI 46 | VAC 21 | *Senegalia* *senegal* (99.85%) NC045513 | *Vachelia* *tortilis* subsp. *raddiana* (99.85%) KY100266 | *Vachelia* *tortilis* subsp. *raddiana* (99.85%) KY100265 |
| ROI 46 | VAC 22 | *Senegalia* *senegal* (94.65%) NC045513 | *Vachelia* *tortilis* subsp. *raddiana* (94.65%) KY100266 | *Vachelia* *tortilis* subsp. *raddiana* (94.65%) KY100265 |
| ROI 46 | VAC 23 | *Senegalia* *senegal* (99.86%) NC045513 | *Vachelia* *tortilis* subsp. *raddiana* (99.86%) KY100266 | *Vachelia* *tortilis* subsp. *raddiana* (99.86%) KY100265 |
| ROI 46 | VAC 24 | *Senegalia* *senegal* (99.86%) NC045513 | *Vachelia* *tortilis* subsp. *raddiana* (99.86%) KY100266 | *Vachelia* *tortilis* subsp. *raddiana* (99.86%) KY100265 |
| ROI 46 | VAC 25 | *Senegalia* *senegal* (99.85%) NC045513 | *Vachelia* *tortilis* subsp. *raddiana* (99.85%) KY100266 | *Vachelia* *tortilis* subsp. *raddiana* (99.85%) KY100265 |
| ROI 47 | VAC 26 | *Senegalia* *senegal* (99.71%) NC045513 | *Vachelia* *tortilis* subsp. *raddiana* (99.71%) KY100266 | *Vachelia* *tortilis* subsp. *raddiana* (99.71%) KY100265 |
| ROI 47 | VAC 27 | *Senegalia* *senegal* (99.86%) NC045513 | *Vachelia* *tortilis* subsp. *raddiana* (99.86%) KY100266 | *Vachelia* *tortilis* subsp. *raddiana* (99.86%) KY100265 |
| ROI 47 | VAC 28 | *Senegalia* *senegal* (99.44%) NC045513 | *Vachelia* *tortilis* subsp. *raddiana* (99.44%) KY100266 | *Vachelia* *tortilis* subsp. *raddiana* (99.44%) KY100265 |
| ROI 47 | VAC 29 | *Senegalia* *senegal* (99.85%) NC045513 | *Vachelia* *tortilis* subsp. *raddiana* (99.85%) KY100266 | *Vachelia* *tortilis* subsp. *raddiana* (99.85%) KY100265 |
| ROI 47 | VAC 30 | *Senegalia* *senegal* (99.85%) NC045513 | *Vachelia* *tortilis* subsp. *raddiana* (99.85%) KY100266 | *Vachelia* *tortilis* subsp. *raddiana* (99.85%) KY100265 |
| ROI 48 | VAC 31 | *Senegalia* *senegal* (99.86%) NC045513 | *Vachelia* *tortilis* subsp. *raddiana* (99.86%) KY100266 | *Vachelia* *tortilis* subsp. *raddiana* (99.86%) KY100265 |
| ROI 48 | VAC 32 | *Senegalia* *senegal* (99.86%) NC045513 | *Vachelia* *tortilis* subsp. *raddiana* (99.86%) KY100266 | *Vachelia* *tortilis* subsp. *raddiana* (99.86%) KY100265 |
| ROI 48 | VAC 33 | *Senegalia* *senegal* (99.85%) NC045513 | *Vachelia* *tortilis* subsp. *raddiana* (99.85%) KY100266 | *Vachelia* *tortilis* subsp. *raddiana* (99.85%) KY100265 |
| ROI 48 | VAC 34 | *Senegalia* *senegal* (99.86%) NC045513 | *Vachelia* *tortilis* subsp. *raddiana* (99.86%) KY100266 | *Vachelia* *tortilis* subsp. *raddiana* (99.86%) KY100265 |
| ROI 48 | VAC 35 | *Senegalia* *senegal* (99.86%) NC045513 | *Vachelia* *tortilis* subsp. *raddiana* (99.86%) KY100266 | *Vachelia* *tortilis* subsp. *raddiana* (99.86%) KY100265 |
| ROI 49 | VAC 36 | *Senegalia* *senegal* (99.55%) NC045513 | *Vachelia* *tortilis* subsp. *raddiana* (99.55%) KY100266 | *Vachelia* *tortilis* subsp. *raddiana* (99.55%) KY100265 |
| ROI 49 | VAC 37 | *Senegalia* *senegal* (94.65%) NC045513 | *Vachelia* *tortilis* subsp. *raddiana* (94.65%) KY100266 | *Vachelia* *tortilis* subsp. *raddiana* (94.65%) KY100265 |
| ROI 49 | VAC 38 | *Senegalia* *senegal* (99.86%) NC045513 | *Vachelia* *tortilis* subsp. *raddiana* (99.86%) KY100266 | *Vachelia* *tortilis* subsp. *raddiana* (99.86%) KY100265 |
| ROI 49 | VAC 39 | *Senegalia* *senegal* (99.72%) NC045513 | *Vachelia* *tortilis* subsp. *raddiana* (99.72%) KY100266 | *Vachelia* *tortilis* subsp. *raddiana* (99.72%) KY100265 |
| ROI 49 | VAC 40 | *Senegalia* *senegal* (99.86%) NC045513 | *Vachelia* *tortilis* subsp. *raddiana* (99.86%) KY100266 | *Vachelia* *tortilis* subsp. *raddiana* (99.86%) KY100265 |
