# Supplementary material for: Ecological aspects and relationships of the emblematic Vachellia spp. exposed to anthropic pressures and parasitism in natural hyper-arid ecosystems: ethnobotanical elements, morphology, and biological nitrogen fixation
Source: Planta. 2024 Apr 25;259(6):132. doi: 10.1007/s00425-024-04407-0 (PMC11045644; doi:10.1007/s00425-024-04407-0)
Supplement: Supplementary file 13 — Supplementary file13 (DOCX 14 KB) [file 425_2024_4407_MOESM13_ESM.docx]

**Table S6** Correlation matrix of the characteristics of plants sampled in AlUla region, including leguminous species, parasites and reference species. Non-parametric Spearman tests were used for the correlation analyses (adjusted with the Bonferroni method). The results are given as *rho* values (in the lower left part of the matrix), and associated significance level (upper right part of the matrix). Correlation significance codes: NS if *P* > 0.05; ***** if *P* < 0.05; ****** if *P* < 0.01; ******* if *P* < 0.001. Significant correlations are highlighted in bold

|  | **δ^15^N** | **δ^13^C** | **%N** | **%C** | **C/N ratio** |
| --- | --- | --- | --- | --- | --- |
| **δ^15^N** |  | ******* | NS | ******* | ******* |
| **δ^13^C** | **0.34** |  | ***** | ******* | NS |
| **%N** | 0.12 | **-0.13** |  | NS | ******* |
| **%C** | -**0.41** | **-0.48** | -0.07 |  | ******* |
| **C/N ratio** | **-0.25** | -0.03 | **-0.93** | **0.37** |  |
