# Supplementary material for: Ecological aspects and relationships of the emblematic Vachellia spp. exposed to anthropic pressures and parasitism in natural hyper-arid ecosystems: ethnobotanical elements, morphology, and biological nitrogen fixation
Source: Planta. 2024 Apr 25;259(6):132. doi: 10.1007/s00425-024-04407-0 (PMC11045644; doi:10.1007/s00425-024-04407-0)
Supplement: Supplementary file 14 — Supplementary file14 (DOCX 20 KB) [file 425_2024_4407_MOESM14_ESM.docx]

**Table S7** Parameters measured in leguminous species, parasites, and reference species in the AlUla region. Results are given as mean ± standard deviation with the significance level according to *post-hoc* tests. For each parameter, the coefficient of variation around the mean (CV) is expressed in %, and calculated according to the following formula: CV = standard deviation / mean × 100

| **Part 1: N and C contents, with C/N ratio.** | | | | | | | |
| --- | --- | --- | --- | --- | --- | --- | --- |
| **Species** | ***n*** | **Nitrogen (%)** | **CV** | **Carbon (%)** | **CV** | **C/N ratio** | **CV** |
| *Atriplex coriacea* | 10 | 1.92 ± 0.24 bcd | 13 | 30.9 ± 1.2 gh | 4 | 19.0 ± 2.2 c | 12 |
| *Brachypodium retusum* | 4 | 1.35 ± 0.31 de | 23 | 41.3 ± 0.3 ef | 1 | 37.0 ± 7.4 bc | 20 |
| *Citrullus colocynthis* | 15 | 2.59 ± 0.77 abc | 30 | 42.2 ± 2.3 e | 5 | 20.8 ± 6.8 c | 33 |
| *Ephedra aphylla* | 9 | 1.85 ± 0.63 bcd | 34 | 42.3 ± 0.5 de | 1 | 31.3 ± 16.9 bc | 54 |
| *Fagonia bruguieri* | 3 | 2.56 ± 0.37 abcd | 14 | 39.2 ± 3.1 ef | 8 | 18.2 ± 3.3 c | 18 |
| *Haloxylon salicornicum* | 28 | 2.77 ± 0.71 a | 26 | 38.8 ± 1.5 f | 4 | 17.5 ± 4.8 c | 27 |
| *Lycium shawii* | 22 | 1.79 ± 0.75 cd | 42 | 45.1 ± 3.6 bcd | 8 | 35.1 ± 19.0 bc | 54 |
| *Ochradenus baccatus* | 20 | 1.53 ± 0.54 d | 35 | 45.6 ± 2.6 bc | 6 | 39.1 ± 15.1 b | 39 |
| *Panicum turgidum* | 19 | 0.76 ± 0.27 e | 36 | 43.3 ± 3.4 cde | 8 | 75.9 ± 32.5 a | 43 |
| *Plicosepalus acaciae* | 11 | 1.49 ± 0.32 de | 21 | 46.0 ± 1.9 b | 4 | 35.0 ± 10.6 bc | 30 |
| *Retama raetam* | 23 | 2.25 ± 0.30 abcd | 13 | 50.3 ± 1.5 a | 3 | 26.6 ± 4.2 bc | 16 |
| *Salsola baryosma* | 10 | 2.70 ± 1.13 ab | 42 | 34.1 ± 2.2 g | 6 | 19.6 ± 13.8 c | 70 |
| *Senna italica* | 6 | 2.67 ± 1.12 abc | 42 | 43.9 ± 2.3 bcde | 5 | 22.9 ± 11.3 c | 49 |
| *Suaeda vermiculata* | 3 | 2.13 ± 0.23 abcd | 11 | 26.4 ± 2.1 h | 8 | 14.5 ± 0.4 c | 3 |
| *Vachellia* spp. | 40 | 2.39 ± 0.29 abcd | 12 | 46.4 ± 1.6 b | 3 | 23.0 ± 2.9 c | 13 |
|  |  |  |  |  |  |  |  |
| **Part 2: C and N isotopic signatures** | | | | | | | |
| **Species** | ***n*** | **δ^15^N (‰)** | **CV** | **δ^13^C (‰)** | **CV** |  |  |
| *Atriplex coriacea* | 10 | 5.66 ± 2.19 ab | 39 | -13.6 ± 0.6 d | 4 |  |  |
| *Brachypodium retusum* | 4 | 3.39 ± 2.45 bc | 72 | -15.1 ± 0.3 d | 2 |  |  |
| *Citrullus colocynthis* | 15 | 4.44 ± 1.93 bc | 43 | -27.2 ± 1.0 bc | 4 |  |  |
| *Ephedra aphylla* | 9 | 3.35 ± 1.71 bc | 51 | -25.9 ± 1.3 c | 5 |  |  |
| *Fagonia bruguieri* | 3 | 5.58 ± 3.08 abc | 55 | -28.3 ± 0.9 abc | 3 |  |  |
| *Haloxylon salicornicum* | 28 | 7.54 ± 1.99 a | 26 | -14.2 ± 0.7 d | 5 |  |  |
| *Lycium shawii* | 22 | 4.46 ± 2.31 bc | 52 | -26.8 ± 2.7 c | 10 |  |  |
| *Ochradenus baccatus* | 20 | 6.27 ± 2.36 ab | 38 | -27.6 ± 1.2 bc | 4 |  |  |
| *Panicum turgidum* | 19 | 3.86 ± 2.69 bc | 70 | -14.0 ± 1.2 d | 9 |  |  |
| *Plicosepalus acaciae* | 11 | 2.09 ± 2.67 bc | 127 | -30.6 ± 1.2 a | 4 |  |  |
| *Retama raetam* | 23 | 1.57 ± 1.38 c | 88 | -26.6 ± 1.5 c | 6 |  |  |
| *Salsola baryosma* | 10 | 6.05 ± 1.60 ab | 26 | -14.8 ± 1.4 d | 9 |  |  |
| *Senna italica* | 6 | 2.35 ± 2.05 bc | 87 | -29.4 ± 1.3 ab | 4 |  |  |
| *Suaeda vermiculata* | 3 | 6.88 ± 2.80 ab | 41 | -15.7 ± 0.6 d | 4 |  |  |
| *Vachellia* spp. | 40 | 2.73 ± 2.23 bc | 82 | -28.0 ± 1.0 bc | 4 |  |  |
