# Supplementary material for: Ecological aspects and relationships of the emblematic Vachellia spp. exposed to anthropic pressures and parasitism in natural hyper-arid ecosystems: ethnobotanical elements, morphology, and biological nitrogen fixation
Source: Planta. 2024 Apr 25;259(6):132. doi: 10.1007/s00425-024-04407-0 (PMC11045644; doi:10.1007/s00425-024-04407-0)
Supplement: Supplementary file 15 — Supplementary file15 (DOCX 14 KB) [file 425_2024_4407_MOESM15_ESM.docx]

**Table S8** Correlation matrix of numerical parameters associated with *Vachellia* tissues, including nitrogen fixation (%Ndfa), N and C isotopic signatures (δ^15^N and δ^13^C), N and C contents (in %) and C/N ratio. Non-parametric Spearman tests were used for the correlation analyses (adjusted with the Bonferroni method). The results are given as *rho* values (in the lower left part of the matrix), and associated significance level (upper right part of the matrix). Correlation significance codes: NS if *P* > 0.05; ***** if *P* < 0.05; ****** if *P* < 0.01; ******* if *P* < 0.001. Significant correlations are highlighted in bold

|  | **%Ndfa** | **δ^15^N** | **δ^13^C** | **N%** | **C%** | **C/N ratio** |
| --- | --- | --- | --- | --- | --- | --- |
| **%Ndfa** |  | ******* | ******* | ******* | NS | ******* |
| **δ^15^N** | **-0.94** |  | ***** | ******* | NS | ******* |
| **δ^13^C** | **0.31** | **-0.18** |  | NS | NS | NS |
| **N%** | **-0.35** | **0.38** | -0.17 |  | ***** | ******* |
| **C%** | -0.17 | 0.15 | -0.11 | **0.20** |  | NS |
| **C/N ratio** | **0.34** | **-0.37** | 0.16 | **-1.00** | -0.14 |  |
