# Supplementary material for: Ecological aspects and relationships of the emblematic Vachellia spp. exposed to anthropic pressures and parasitism in natural hyper-arid ecosystems: ethnobotanical elements, morphology, and biological nitrogen fixation
Source: Planta. 2024 Apr 25;259(6):132. doi: 10.1007/s00425-024-04407-0 (PMC11045644; doi:10.1007/s00425-024-04407-0)
Supplement: Supplementary file 16 — Supplementary file16 (DOCX 16 KB) [file 425_2024_4407_MOESM16_ESM.docx]

**Table S9** Correlation matrix of numerical parameters associated wwith *Vachellia* trees, including nitrogen fixation (%Ndfa), N and C isotopic signatures (δ^15^N and δ^13^C), N and C contents (%), C/N ratio and morphological traits (health status, tree and trunk heights, and trunk diameter at breast height). The data used for this analysis are based on leaf samples (*n* = 5 replicates × 8 ROIs = 40 measures). Non-parametric Spearman tests were used for the correlation analyses (adjusted with the Bonferroni method). The results are given as *rho* values (in the lower left part of the matrix) and associated significance level (upper right part of the matrix). Correlation significance codes: NS if *P* > 0.05; ***** if *P* < 0.05; ****** if *P* < 0.01; ******* if *P* < 0.001. Significant correlations are highlighted in bold

|  | **%Ndfa** | **δ^15^N** | **δ^13^C** | **N%** | **C%** | **C/N ratio** | **Tree height** | **Trunk height** | **Trunk DBH** |
| --- | --- | --- | --- | --- | --- | --- | --- | --- | --- |
| **%Ndfa** |  | ******* | ***** | NS | NS | NS | ***** | NS | ***** |
| **δ^15^N** | **-0.91** |  | NS | NS | NS | NS | ***** | NS | ******* |
| **δ^13^C** | **0.33** | -0.14 |  | NS | NS | NS | ***** | ****** | NS |
| **N%** | 0.09 | -0.01 | 0.19 |  | NS | ******* | NS | NS | NS |
| **C%** | -0.22 | 0.21 | -0.14 | -0.03 |  | NS | NS | NS | NS |
| **C/N ratio** | -0.15 | 0.07 | -0.19 | **-0.97** | 0.25 |  | NS | NS | NS |
| **Tree height** | **-0.39** | **0.36** | **-0.32** | 0.01 | 0.25 | 0.05 |  | ******* | ****** |
| **Trunk height** | -0.25 | 0.21 | **-0.45** | -0.01 | 0.24 | 0.04 | **0.65** |  | NS |
| **Trunk DBH** | **-0.39** | **0.53** | 0.02 | 0.04 | 0.21 | 0.01 | **0.44** | 0.16 |  |
