# Supplementary material for: Ecological aspects and relationships of the emblematic Vachellia spp. exposed to anthropic pressures and parasitism in natural hyper-arid ecosystems: ethnobotanical elements, morphology, and biological nitrogen fixation
Source: Planta. 2024 Apr 25;259(6):132. doi: 10.1007/s00425-024-04407-0 (PMC11045644; doi:10.1007/s00425-024-04407-0)
Supplement: Supplementary file 18 — Supplementary file18 (DOCX 17 KB) [file 425_2024_4407_MOESM18_ESM.docx]

**Table S11** Correlation matrix of numerical parameters associated with the parasite (*P*. *acaciae*) and its host (*Vachellia*), including N and C isotopic signatures (δ^15^N and δ^13^C), N and C contents (%), and the C/N ratio of each organism. Multiple non-parametric Spearman tests were used for the correlation analyses (adjusted with the Bonferroni method). The results are given as *rho* values (in the lower left part of the matrix), and associated significance level (upper right part of the matrix, where NS corresponds to *P* > 0.05). Intraspecific correlations are highlighted in yellow/orange and interspecific correlations in green.

|  | **Host**  **δ^15^N** | **Host**  **δ^13^C** | **Host %N** | **Host %C** | **Host C/N ratio** | **Host %NDFA** | **Parasite δ^15^N** | **Parasite δ^13^C** | **Parasite %N** | **Parasite %C** | **Parasite C/N ratio** | **Parasite %NDFA** |
| --- | --- | --- | --- | --- | --- | --- | --- | --- | --- | --- | --- | --- |
| **Host**  **δ^15^N** |  | NS | NS | NS | NS | ******* | ******* | NS | ******* | NS | ******* | ******* |
| **Host**  **δ^13^C** | -0.47 |  | NS | NS | NS | NS | NS | NS | NS | NS | ***** | NS |
| **Host %N** | -0.21 | 0.27 |  | NS | ******* | NS | NS | NS | NS | NS | NS | NS |
| **Host %C** | 0.26 | 0.19 | 0.25 |  | NS | NS | NS | NS | NS | NS | NS | NS |
| **Host C/N ratio** | 0.41 | -0.29 | **-0.95** | 0.03 |  | NS | NS | NS | NS | NS | NS | NS |
| **Host %NDFA** | **-0.93** | 0.51 | 0.31 | -0.33 | -0.48 |  | ******* | NS | ******* | NS | ******* | ******* |
| **Parasite δ^15^N** | **0.89** | -0.13 | -0.08 | 0.50 | 0.32 | **-0.86** |  | NS | ***** | NS | ***** | ******* |
| **Parasite δ^13^C** | 0.18 | 0.04 | -0.13 | 0.18 | 0.13 | -0.18 | 0.12 |  | NS | NS | NS | NS |
| **Parasite %N** | **0.76** | -0.41 | -0.32 | 0.09 | 0.38 | **-0.76** | **0.57** | 0.58 |  | NS | ******* | ***** |
| **Parasite %C** | -0.14 | 0.03 | 0.42 | 0.63 | -0.30 | 0.06 | 0.02 | 0.34 | -0.10 |  | NS | NS |
| **Parasite C/N ratio** | **-0.77** | **0.45** | 0.42 | -0.03 | -0.48 | **0.76** | **-0.55** | -0.58 | **-0.98** | 0.15 |  | ***** |
| **Parasite %NDFA** | **-0.83** | 0.37 | 0.02 | -0.57 | -0.24 | **0.92** | **-0.86** | -0.07 | **-0.59** | -0.14 | **0.54** |  |
