# Supplementary material for: Ecological aspects and relationships of the emblematic Vachellia spp. exposed to anthropic pressures and parasitism in natural hyper-arid ecosystems: ethnobotanical elements, morphology, and biological nitrogen fixation
Source: Planta. 2024 Apr 25;259(6):132. doi: 10.1007/s00425-024-04407-0 (PMC11045644; doi:10.1007/s00425-024-04407-0)
Supplement: Supplementary file 19 — Supplementary file19 (DOCX 14 KB) [file 425_2024_4407_MOESM19_ESM.docx]

**Table S12** *Retama* *raetam* characteristics across locations. Results are expressed as means ± standard deviation. Kruskal-Wallis tests, followed by Dunn tests for multiple means comparisons. Significant different means levels (at *P* < 0.05) are indicated with letters (‘a’, ‘b’, etc.). Treatments with the same letter are considered as similar (e.g. the ‘ab’level is not significantly different from the ‘a’ or ‘b’ level)

|  | **Djabal Abu Oud** *n* = 4 | **Madakhil** *n* = 10 | **Sharaan** *n* = 9 |
| --- | --- | --- | --- |
| **Nitrogen (%)** | 2.03 ± 0.28 a | 2.25 ± 0.33 a | 2.35 ± 0.27 a |
| **Carbon (%)** | 49.8 ± 0.8 ab | 51.5 ± 1.4 a | 49.2 ± 0.9 b |
| **C/N ratio** | 29.1 ± 3.7 a | 27.3 ± 5.2 a | 24.7 ± 2.6 a |
| **δ^15^N (‰)** | 0.67 ± 0.50 a | 2.02 ± 1.46 a | 1.42 ± 1.52 a |
| **δ^13^C (‰)** | -26.2 ± 1.0 b | -25.4 ± 0.8 b | -28.1 ± 1.0 a |
| **%Ndfa** | 79.5 ± 15.6 a | 64.0 ± 24.8 a | 60.3 ± 40.4 a |
