# Supplementary material for: Ecological aspects and relationships of the emblematic Vachellia spp. exposed to anthropic pressures and parasitism in natural hyper-arid ecosystems: ethnobotanical elements, morphology, and biological nitrogen fixation
Source: Planta. 2024 Apr 25;259(6):132. doi: 10.1007/s00425-024-04407-0 (PMC11045644; doi:10.1007/s00425-024-04407-0)
Supplement: Supplementary file 20 — Supplementary file20 (DOCX 14 KB) [file 425_2024_4407_MOESM20_ESM.docx]

**Table S13** Correlation matrix of numerical parameters associated with *Vachellia* and *R*. *raetam* trees, including N and C isotopic signatures (δ^15^N and δ^13^C), N and C contents (%), C/N ratio, and nitrogen fixation (%Ndfa). The data used for this analysis were based only on leaf samples (*n* = 23 *R*. *raetam* + 25 *Vachellia* replicates on 5 ROIs = 48 measures). Non-parametric Spearman tests were used for the correlation analyses (adjusted with the Bonferroni method). The results are given as *rho* values (in the lower left part of the matrix), and associated significance level (upper right part of the matrix). Correlation significance codes: NS if *P* > 0.05; ***** if *P* < 0.05; ****** if *P* < 0.01; ******* if *P* < 0.001. Significant correlations are highlighted in bold

|  | **δ^15^N** | **δ^13^C** | **%N** | **%C** | **C/N ratio** | **%Ndfa** |
| --- | --- | --- | --- | --- | --- | --- |
| **δ^15^N** |  | NS | NS | NS | NS | ******* |
| **δ^13^C** | -0.27 |  | NS | ******* | ***** | ***** |
| **%N** | 0.11 | -0.16 |  | NS | ******* | NS |
| **%C** | -0.03 | **0.61** | -0.05 |  | ****** | NS |
| **C/N ratio** | -0.10 | **0.34** | **-0.92** | **0.39** |  | NS |
| **%Ndfa** | **-0.94** | **0.35** | -0.14 | 0.09 | 0.16 |  |
